# Supplementary material for: Infectious Complications in Injection Drug Use
Source: MedEdPORTAL. 2021 Mar 23;17:11124. doi: 10.15766/mep_2374-8265.11124 (PMC8015638; doi:10.15766/mep_2374-8265.11124)
Supplement: Supplementary file 1 — Facilitator Guide.docxdPre- and Postsurvey.docxInfectious Disease Complications in IDU Workshop.pptxCase 1 and Case 2 Handout.pptxAnswer Key.docx [file mep_2374-8265.11124-s001.zip › A. Facilitator Guide.docx]

**Facilitator’s Guide**

**Appendix A**: Facilitator’s guide for case-based, interactive workshop on infectious complications of injection drug use (IDU)

**Purpose/Goals**

The overall goal of the session is to increase familiarity and comfort in diagnosing and managing several common infectious complications of IDU that students and house staff will encounter in both the inpatient and outpatient setting.

The goal of the small group break-out discussions is to facilitate critical thinking and problem solving among participants, giving all members an impetus to contribute. Small group break outs allow for participants who may not speak up in large group discussion settings to share their thoughts and for all participants to draw on experiences and knowledge of group members to answer the questions. We allowed for use of outside resources for help in answering questions.

**Practical Implementation Advice**

- To save time during the session, the audience can be asked to complete the pre-session survey the night prior to the workshop

- The audience should be divided up into small groups at the start of the workshop with the facilitator specifically delineating these groups. Groups of 3-4 work well for this.

- One of the facilitators should pass out the clinical summary slides for Cases 1 and 2 so that each small group has a reference to the pertinent case data when the questions are displayed. This can also be emailed out ahead of time, especially if the session is virtual.

- A lesson we learned while facilitating the sessions was to be intentional about choosing which small group would lead off discussions when reconvening as a large group. We found that the first question of the small group encounters tended to be more straightforward while the second and third questions involved more application. Rotating which group answered each allowed us to assess the critical thinking of all groups equally.

- Prior to the session, it is helpful to know what methods your institution uses for microbiological identification so as to give practical information to learners about the type of information they receive from the microbiology lab as well as how quickly they receive it (Blood Culture Identification [BCID] vs Matrix-assisted laser desorption/ionization Time of Flight [MALDI TOF]) vs other.

- Similarly, researching your institutional policy on particular formulary medications or preferred treatment regimen for *S aureus* bacteremia prior to the session will allow you to deliver practical content to your learners. This comes up in Case 2 and may vary by institution.

- We found it helpful to speak with a local expert on injection habits and disease trends to ask specifically about what types of interview questions are appropriate and necessary to teach our participants about. We strongly recommend interfacing with a local expert to tailor educational pearls to your specific learners and patient population

- This workshop can be adapted to an entirely virtual experience, hosted on a video-conferencing platform if it cannot be delivered in-person. While we held two sessions in person, the other two occurred virtually. When conducting this workshop virtually, we suggest using a breakout room or other small group generating feature for the appropriate sections. We also found benefit in using the chat feature to elicit participation from all members.

**Case 1:**

This patient has cellulitis from IDU. The first key learning point is that IDU is a risk factor for MRSA skin and soft tissue infections (SSTIs) and that MRSA coverage needs to be added empirically when considering antimicrobial choices (irrespective of purulent vs non-purulent status).^1^ De-escalation is warranted pending clinical course and should be emphasized. The slide about overnight complications reflects the spectrum of severity of SSTIs and discussion in the large group should focus on practical tips on how to respond to these complications (i.e. how to confirm the diagnosis, which consultants to involve and how urgently).

**Case 2**

This patient has an invasive staphylococcal infection, complicated by bacteremia, endocarditis, and ultimately vertebral osteomyelitis. Starting off, we used the microbiology lab report to prime our audience to be concerned with bacteremia or endovascular infection. We used this as an opportunity to ask them specifically about the type of interview questions or physical exam findings they would employ/look for in this patient. This typically brought up the many eponymous findings associated with IE and if time permitted, we gave the odds ratios for a few of the more common ones to demonstrate that these findings are actually helpful. Moving on to the questions, we again stressed the importance of including MRSA coverage when starting an empiric antimicrobial regimen. Our hospital recommends treating *Staphylococcus aureus* bacteremia (SAB) with dual coverage (MRSA-active agent and beta lactam, either cefazolin or nafcillin) until susceptibility testing is back. From there, therapy is de-escalated to a beta lactam alone if MSSA or dual-coverage continued if MRSA given synergistic effects and reduced time to clearance of blood cultures.^2-4^ We realize this practice will not be the same at all institutions and the answer to Question #2 on Slide 14 can be adapted accordingly.

The amount of time spent on the slide detailing the modified Duke’s criteria can vary depending on how the pace of the session is proceeding. It can briefly be touched upon to remind the audience that the tool exists or can include an explanation of the criteria as in the “notes” section. The criteria that our patient meets become highlighted in red with an animation on slide transition.

The subsequent slide (16) can be used as is if your institution has BCID for microbial identification. If not, there is a slide at the end of the PowerPoint which can replace it. Following the diagnosis of IE, it should be emphasized that formal cardiology and ID consultation should be obtained (ID consultation in *Staph aureus* bacteremia leads to decreased mortality).^5^ The culture data provided on slide should prompt the audience to de-escalate antibiotics to a beta lactam as MRSA coverage is no longer needed. Duration can be discussed for left-sided disease, referencing the IDSA/AHA guidelines, underscoring any time course must start from date of first negative culture.^6^ Discussion regarding the use of oral antibiotics in patients with IE may come up here but is actually addressed a few slides down the line. Subsequently, the significant mortality benefit associated with use of beta lactams in MSSA bacteremia over vancomycin is highlighted.

The next part of Case 2 can be cut if time does not allow. In brief, it explores the workup of acute back pain in a patient with endovascular staphylococcal infection. Multiple learning points are touched upon including the differential, practical physical exam pearls, and imaging modalities. Following the diagnosis of vertebral osteomyelitis, discussion of the POET trial can occur pending time. The talking points we found most helpful addressed the demographics and inclusion criteria, pointing out that there were no PWID and very few MRSA isolates despite neither of these being exclusion criteria. We emphasized that POET is a sentinel study and will likely be practice-changing at some point though are not universally generalizable to the present clinical cases.^7^

**Case 3**

Moving to the ambulatory setting, the focus shifts to the screening tests and immunizations that should be offered to PWID. When the small groups are reporting back, a list can be created on a white board or populated on the PowerPoint. Special consideration paid to Hepatitis A immunization given outbreaks among PWID in homeless shelters across the country. The table detailing STI testing can be covered briefly but attention should be drawn to testing patients’ sites of exposure for gonorrhea/chlamydia as well as the ability for patients to self-swab for gonorrhea/chlamydia and vaginitis testing. The other major learning point of this case is regarding eligibility for PrEP and counseling patients about its use. Use is indicated in anyone sharing equipment or engaging in transactional sex. We consulted a local expert about how to have these conversations in a sensitive, non-judgmental way as well as consider local disease trends and current local injection practices and we recommend doing the same at your institution. For example, a suggestion of non-stigmatizing but broad language to use when screening for transactional sex by one of our local experts was to ask, “Do you ever trade sex for money, drugs, or a place to stay?”

**Table 1: Adapting to 60 or 90 Minute Session**

|  | Duration | 60 Minute Session | 90 Minute Session |
| --- | --- | --- | --- |
| Pre-Session Survey | 5 minutes | X | X |
| Introduction | 5 minutes | X | X |
| **Case 1: Cellulitis** | **20 minutes** | X | X |
| **Case 2:** | **35 minutes** |  |  |
| -- Bacteremia/IE | 20 minutes | X | X |
| -- Back Pain/OM | 10 minutes | X | X |
| -- POET Highlights | 5 minutes |  | X |
| **Case 3:** | **15 minutes** |  |  |
| -- Screening | 10 minutes |  | X |
| -- PrEP | 5 minutes |  | X |
| Summary and Closing | 5 minutes | X | X |
| Post-Session Survey | 5 minutes | X | X |

If reducing the duration of the workshop to 60 minutes, we suggest assigning the pre-session survey to be completed prior to the session. We also recommend removing Questions 5 and 6 from the survey as they pertain to Case 3.

**References**

1. Stevens DL, Bisno AL, Chambers HF, et al. Practice guidelines for the diagnosis and management of skin and soft tissue infections: 2014 Update by the Infectious Diseases Society of America. *Clin Infect Dis*. 2014;59(2):147-159. doi:10.1093/cid/ciu296

2. Davis JS, Sud A, O'Sullivan MVN, et al. Combination of Vancomycin and β-Lactam Therapy for Methicillin-Resistant Staphylococcus aureus Bacteremia: A Pilot Multicenter Randomized Controlled Trial. *Clin Infect Dis*. 2016;62(2):173-180. doi:10.1093/cid/civ808

3. Casapao AM, Jacobs DM, Bowers DR, Beyda ND, Dilworth TJ; REACH-ID Study Group. Early Administration of Adjuvant β-Lactam Therapy in Combination with Vancomycin among Patients with Methicillin-Resistant Staphylococcus aureus Bloodstream Infection: A Retrospective, Multicenter Analysis. *Pharmacotherapy*. 2017;37(11):1347-1356. doi:10.1002/phar.2034

4. Truong J, Veillette JJ, Forland SC. Outcomes of Vancomycin plus a β-Lactam versus Vancomycin Only for Treatment of Methicillin-Resistant Staphylococcus aureus Bacteremia. *Antimicrob Agents Chemother*. 2018;62(2):e01554-17. Published 2018 Jan 25. doi:10.1128/AAC.01554-17

5. Sherbuk JE, McManus D, Topal JE, Malinis M. Improved mortality in Staphylococcus aureus bacteremia with the involvement of antimicrobial stewardship team and infectious disease consultation. *Infect Control Hosp Epidemiol*. 2019; 40(8):932-935. doi:10.1017/ice.2019.136

6. Baddour LM, Wilson WR, Bayer AS, et al. Infective Endocarditis in Adults: Diagnosis, Antimicrobial Therapy, and Management of Complications: A Scientific Statement for Healthcare Professionals From the American Heart Association. *Circulation*. 2015;132(15):1435-1486. doi:10.1161/CIR.0000000000000296

7. Iverson K, Ihlemann N, Gill, SU, et. al. Partial Oral versus Intravenous Antibiotic Treatment of Endocarditis. *N Engl J Med*. 2019; 380:415-424. doi: 10.1056/NEJMoa1808312
