# Supplementary material for: Infectious Complications in Injection Drug Use
Source: MedEdPORTAL. 2021 Mar 23;17:11124. doi: 10.15766/mep_2374-8265.11124 (PMC8015638; doi:10.15766/mep_2374-8265.11124)
Supplement: Supplementary file 1 — Facilitator Guide.docxdPre- and Postsurvey.docxInfectious Disease Complications in IDU Workshop.pptxCase 1 and Case 2 Handout.pptxAnswer Key.docx [file mep_2374-8265.11124-s001.zip › C. Infectious Disease Complications in IDU Workshop.pptx]

## Slide 1
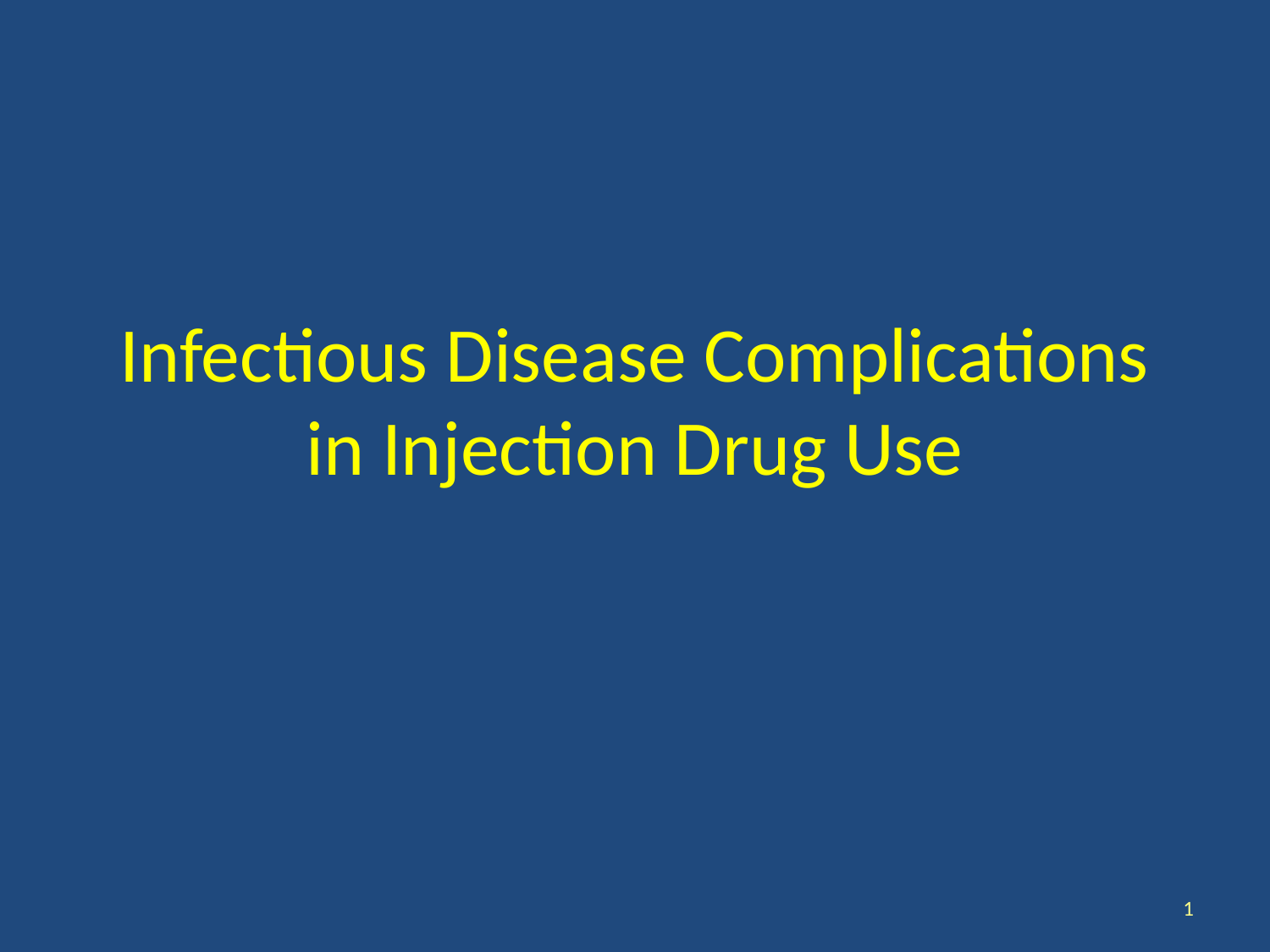

# Infectious Disease Complications in Injection Drug Use
1

## Slide 2
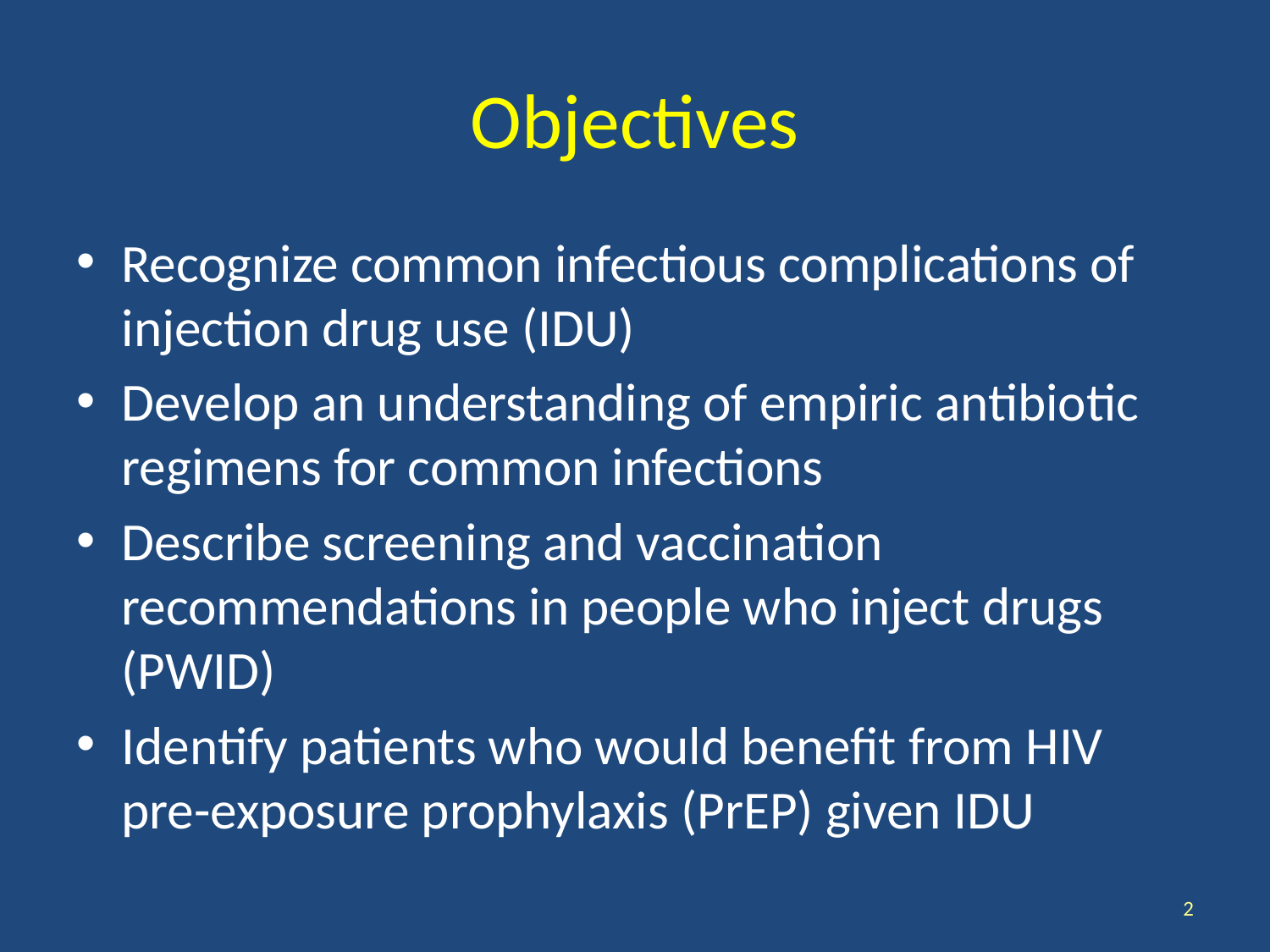

# Objectives
Recognize common infectious complications of injection drug use (IDU)
Develop an understanding of empiric antibiotic regimens for common infections
Describe screening and vaccination recommendations in people who inject drugs (PWID)
Identify patients who would benefit from HIV pre-exposure prophylaxis (PrEP) given IDU
2

## Slide 3
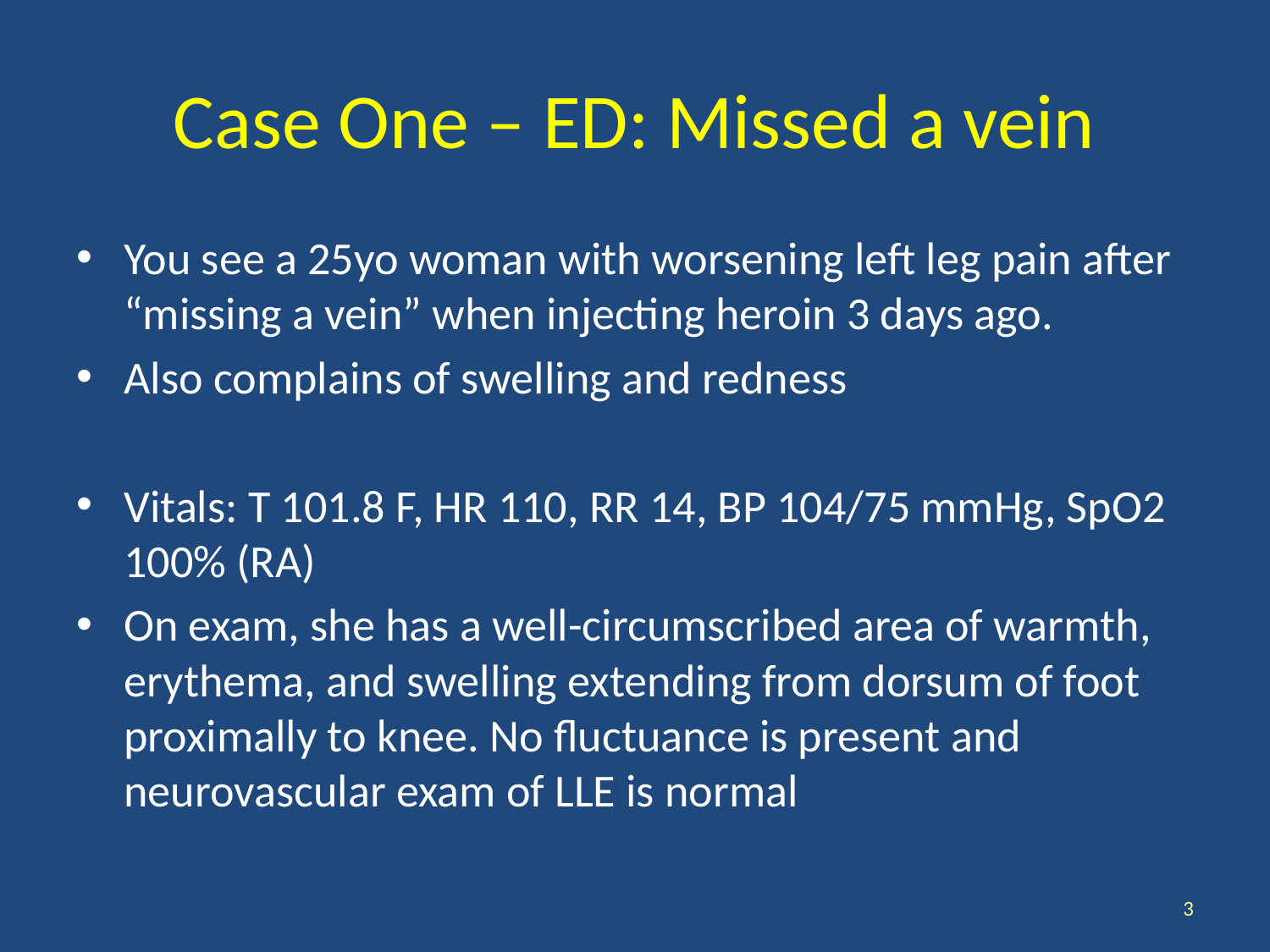

# Case One – ED: Missed a vein
You see a 25yo woman with worsening left leg pain after “missing a vein” when injecting heroin 3 days ago.
Also complains of swelling and redness
Vitals: T 101.8 F, HR 110, RR 14, BP 104/75 mmHg, SpO2 100% (RA)
On exam, she has a well-circumscribed area of warmth, erythema, and swelling extending from dorsum of foot proximally to knee. No fluctuance is present and neurovascular exam of LLE is normal
3

## Slide 4
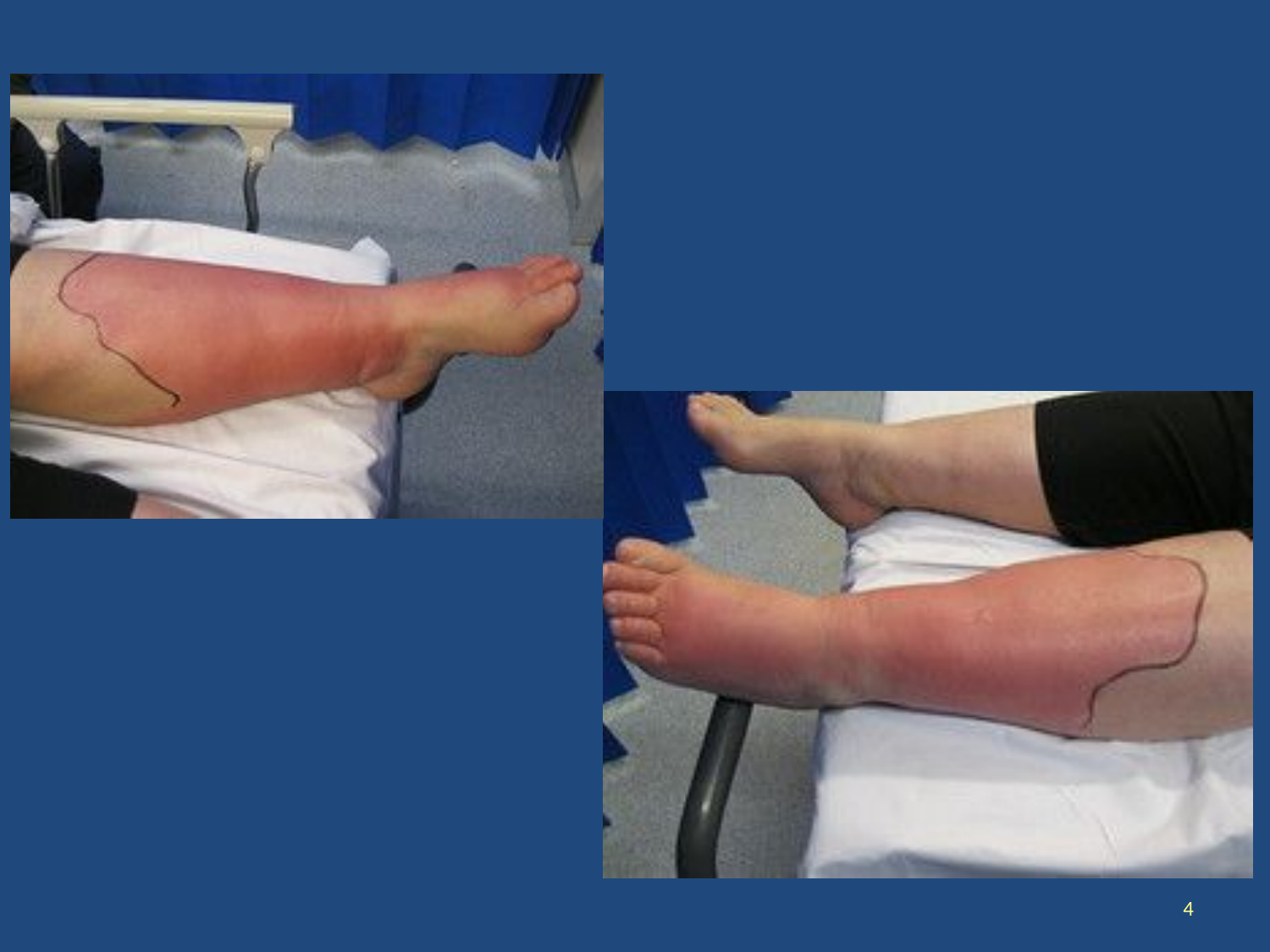

#
4

## Slide 5
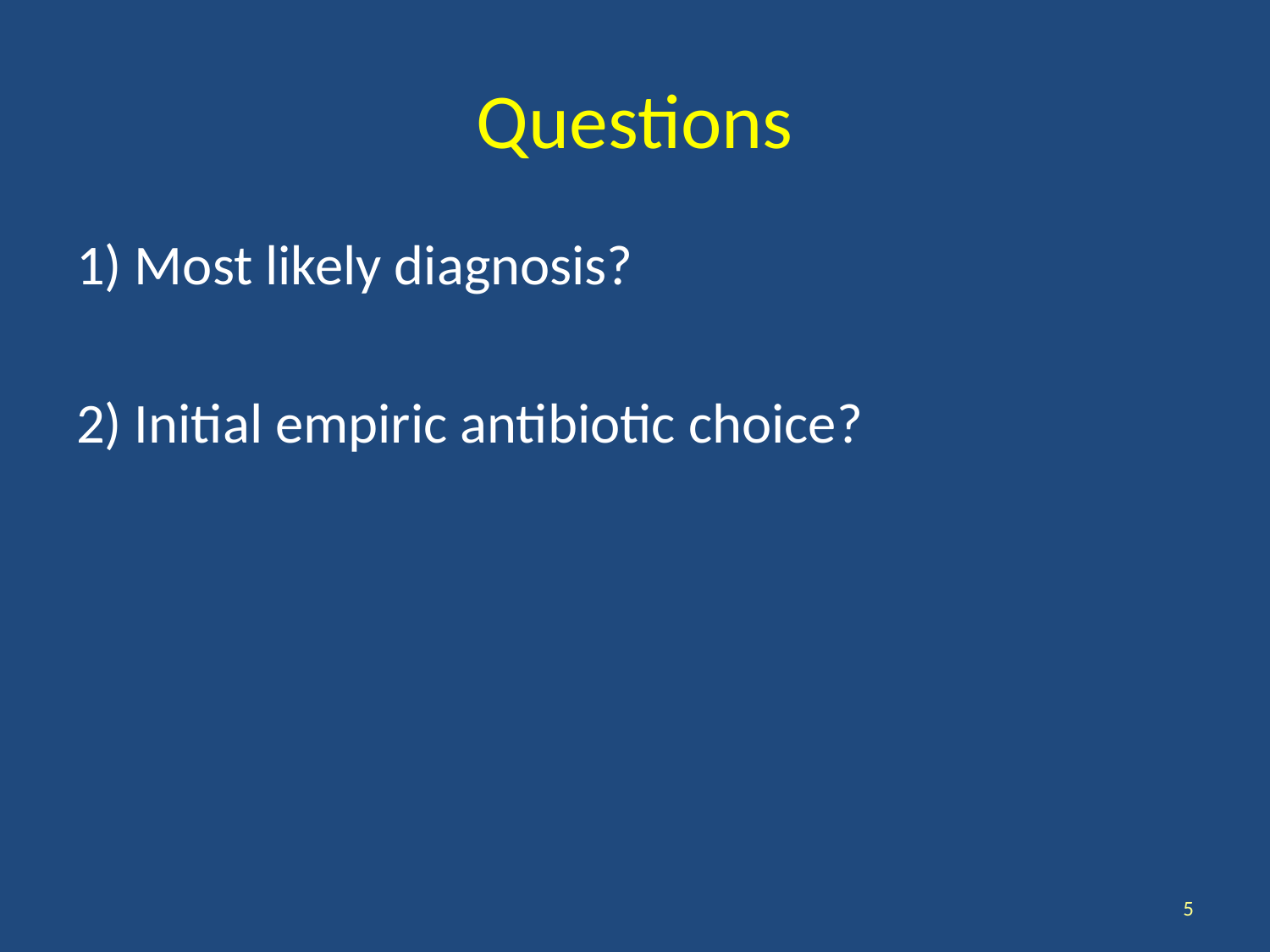

# Questions
1) Most likely diagnosis?
2) Initial empiric antibiotic choice?
5

## Slide 6
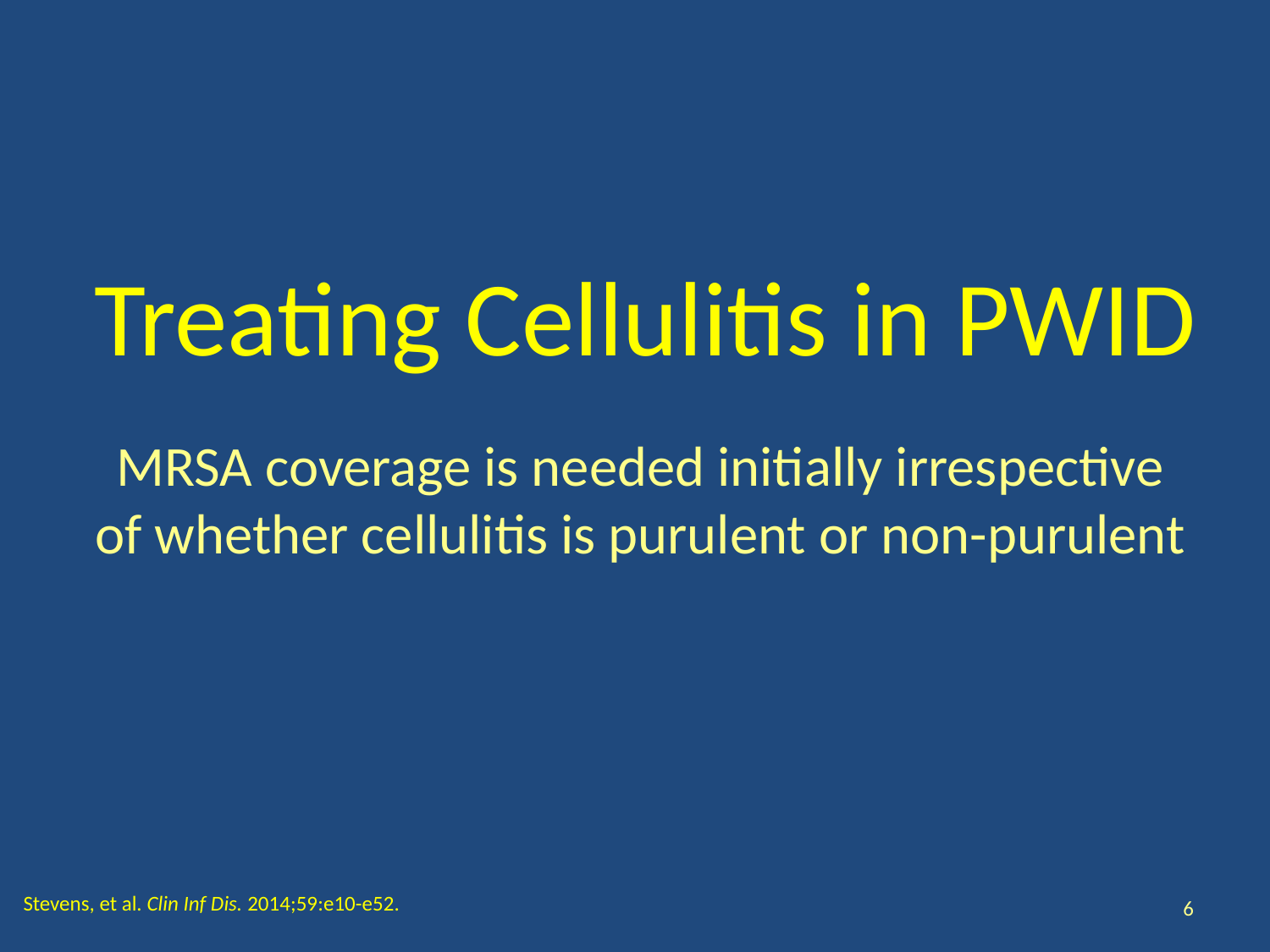

# Treating Cellulitis in PWID
MRSA coverage is needed initially irrespective of whether cellulitis is purulent or non-purulent
Stevens, et al. Clin Inf Dis. 2014;59:e10-e52.
6

## Slide 7
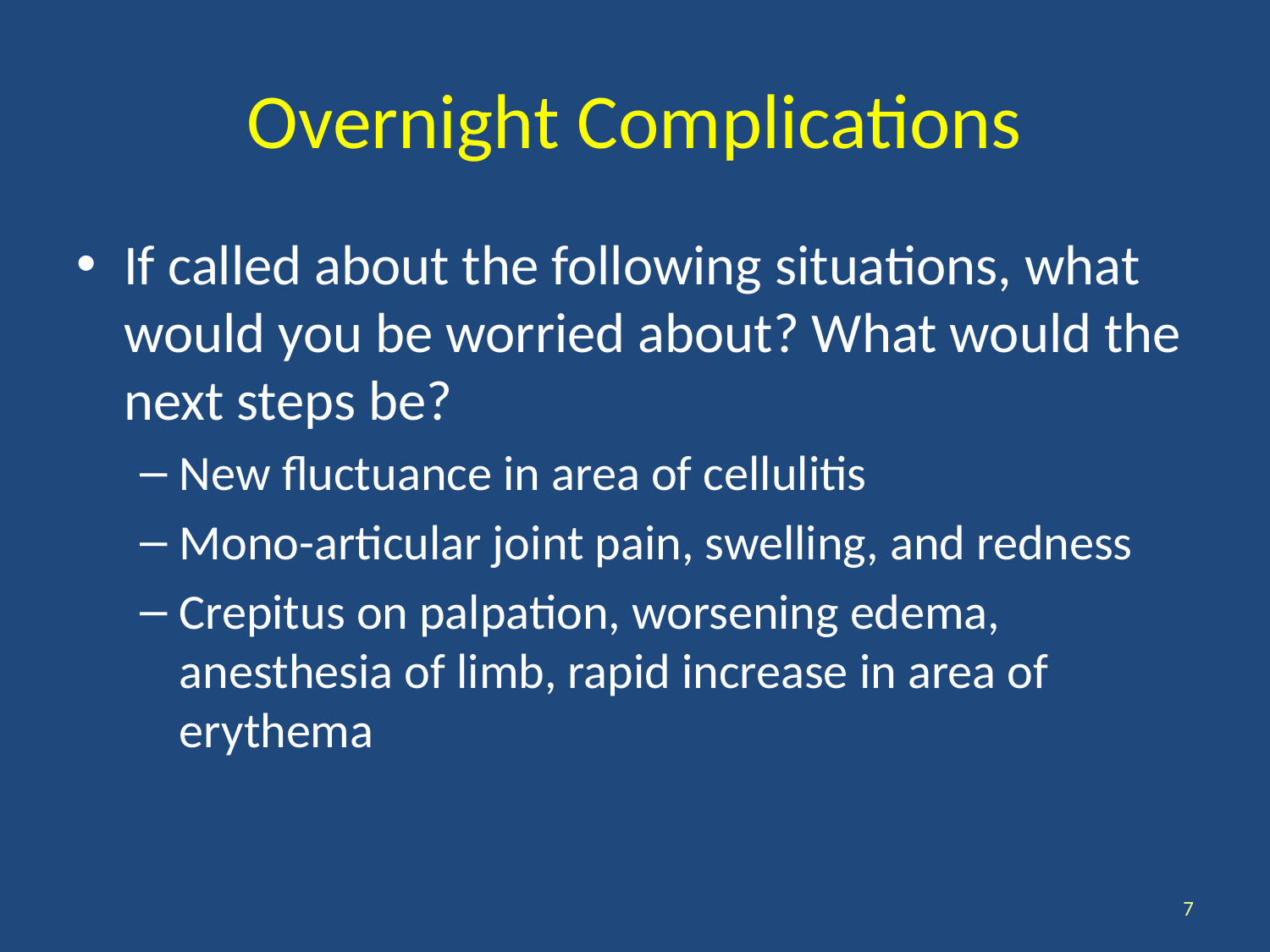

# Overnight Complications
If called about the following situations, what would you be worried about? What would the next steps be?
New fluctuance in area of cellulitis
Mono-articular joint pain, swelling, and redness
Crepitus on palpation, worsening edema, anesthesia of limb, rapid increase in area of erythema
7

## Slide 8
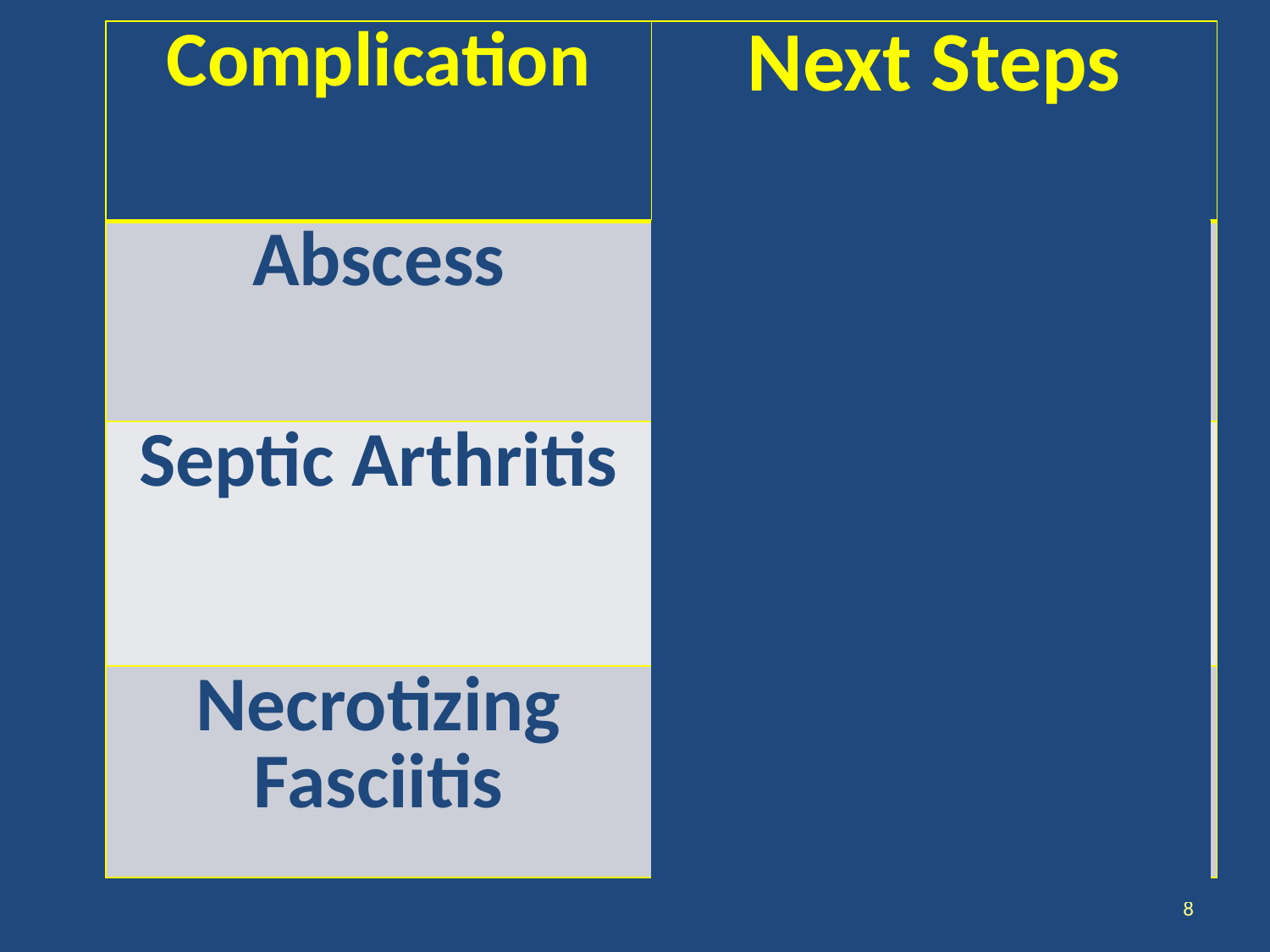

| Complication | Next Steps |
| --- | --- |
| Abscess | Superficial US And Drainage |
| Septic Arthritis | Arthrocentesis with Gram Stain/Culture |
| Necrotizing Fasciitis | STAT surgical consult |
#
8

## Slide 9
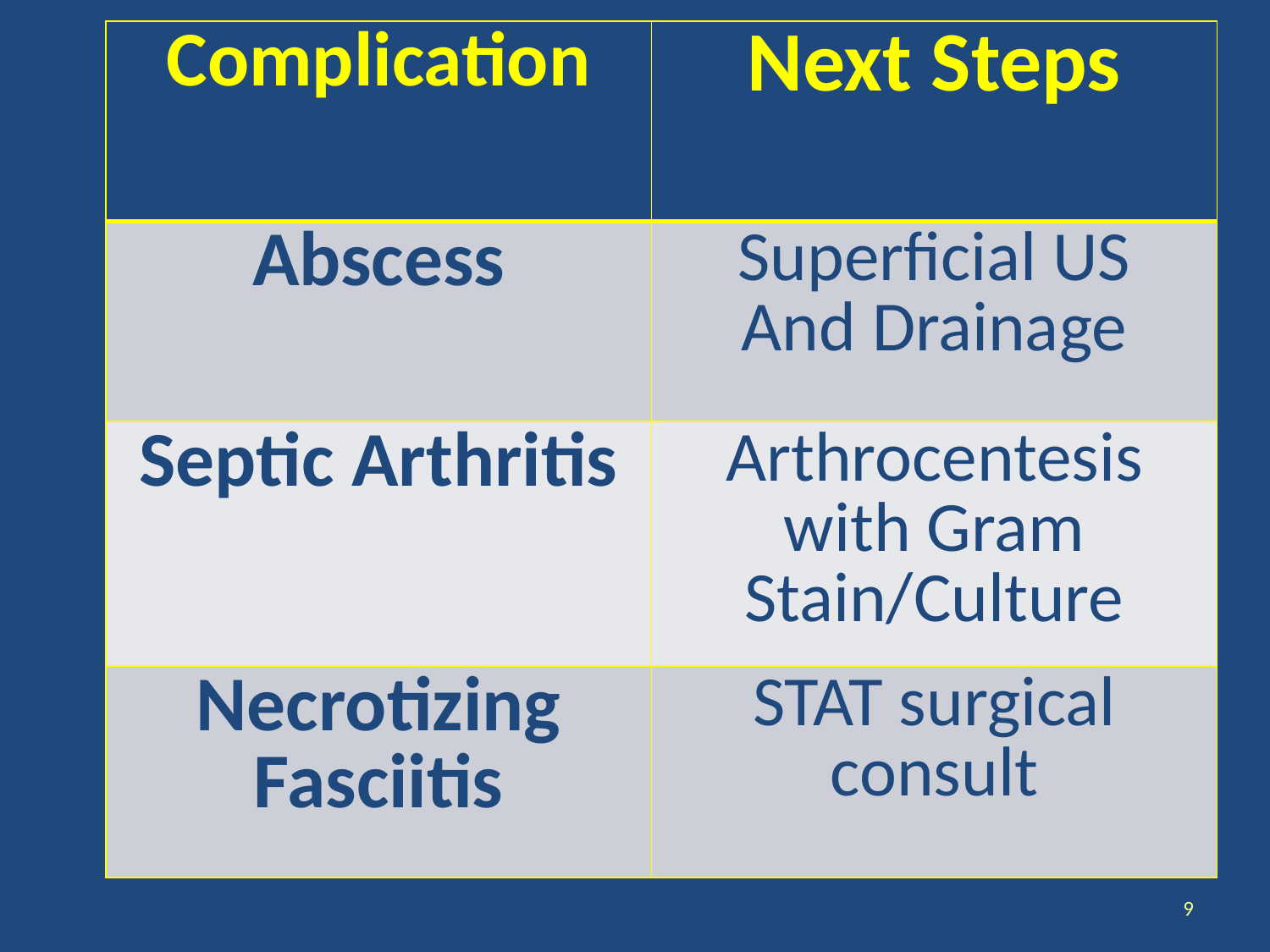

| Complication | Next Steps |
| --- | --- |
| Abscess | Superficial US And Drainage |
| Septic Arthritis | Arthrocentesis with Gram Stain/Culture |
| Necrotizing Fasciitis | STAT surgical consult |
#
9

## Slide 10
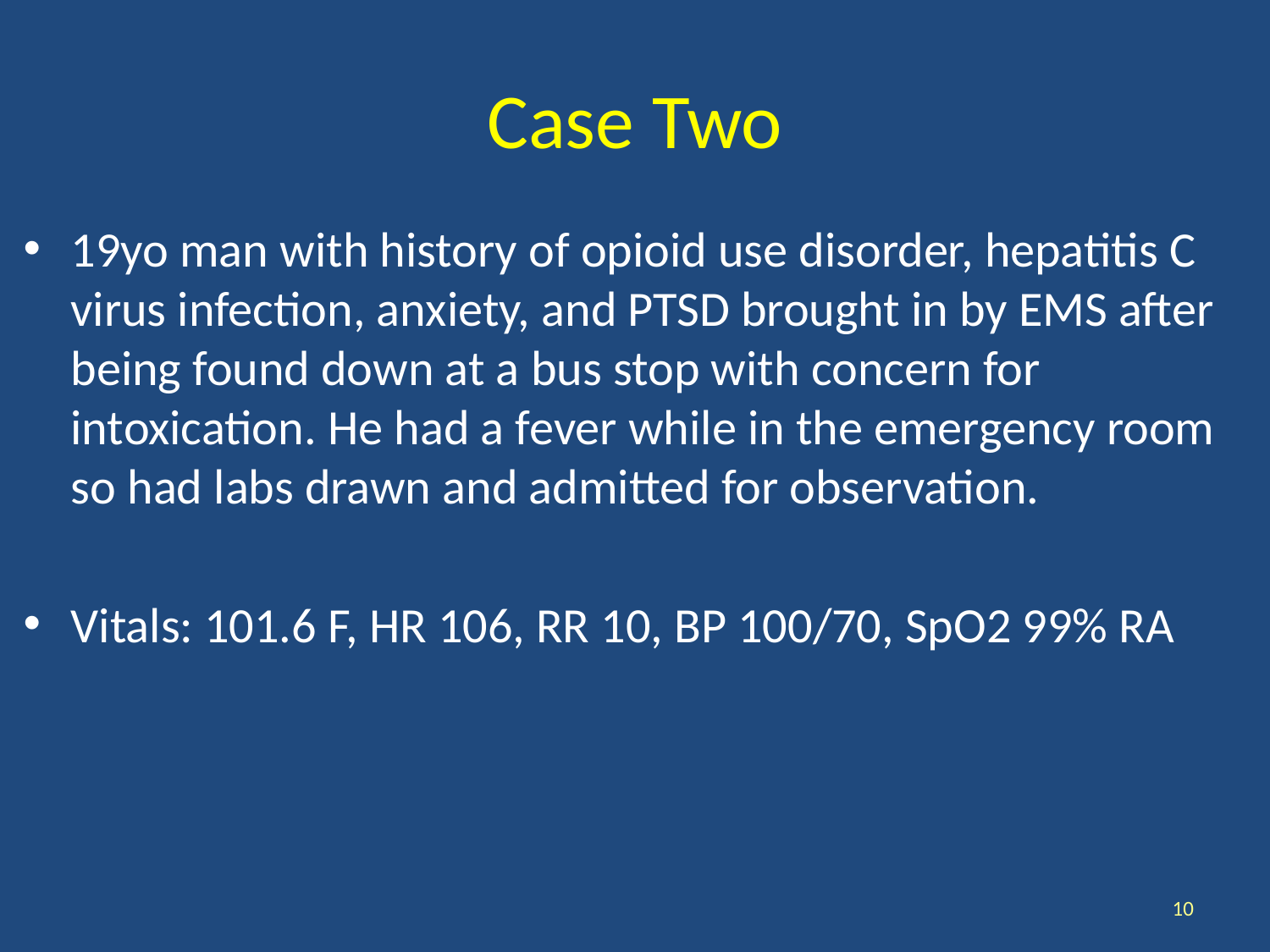

# Case Two
19yo man with history of opioid use disorder, hepatitis C virus infection, anxiety, and PTSD brought in by EMS after being found down at a bus stop with concern for intoxication. He had a fever while in the emergency room so had labs drawn and admitted for observation.
Vitals: 101.6 F, HR 106, RR 10, BP 100/70, SpO2 99% RA
10

## Slide 11
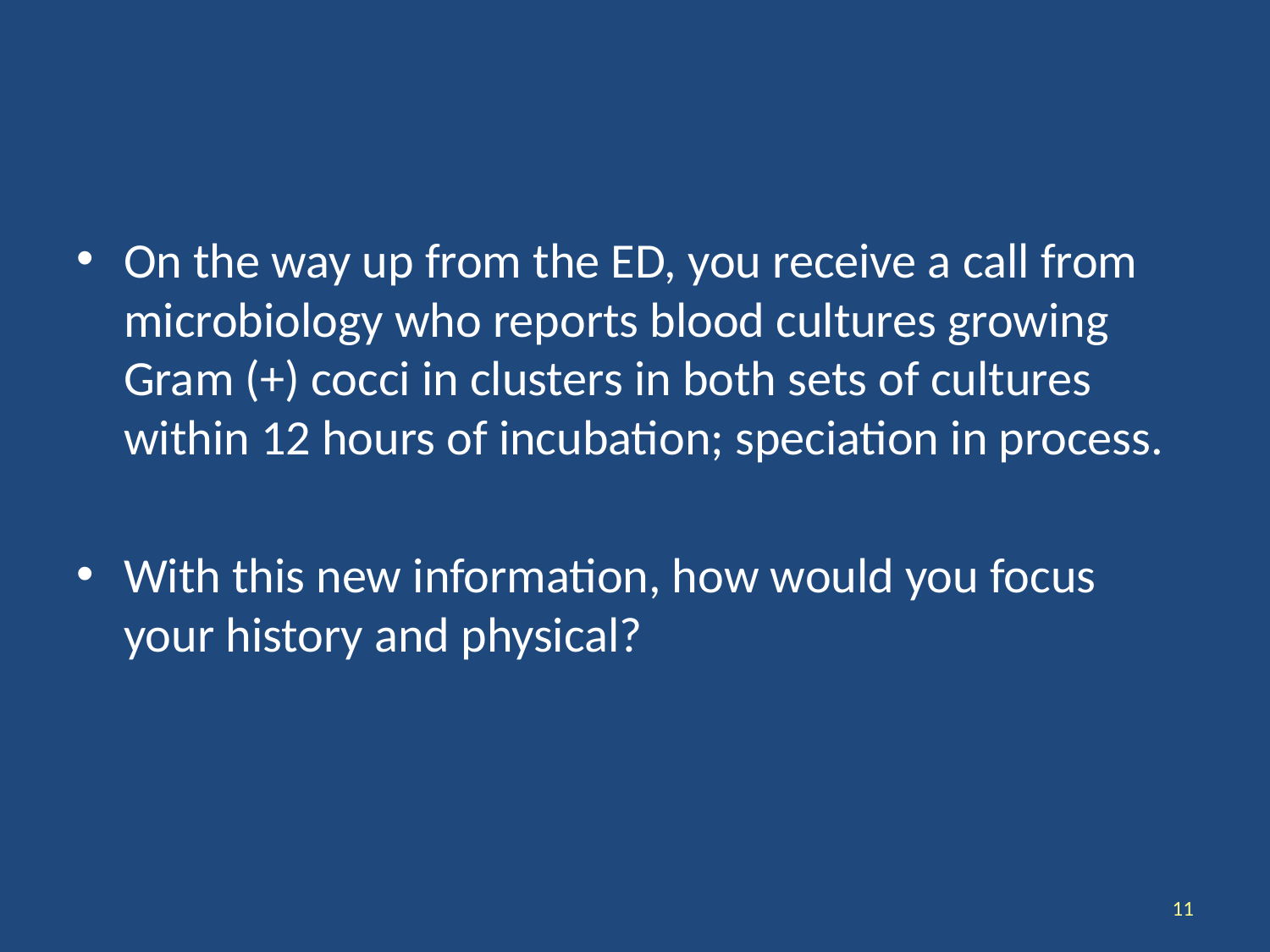

#
On the way up from the ED, you receive a call from microbiology who reports blood cultures growing Gram (+) cocci in clusters in both sets of cultures within 12 hours of incubation; speciation in process.
With this new information, how would you focus your history and physical?
11

## Slide 12
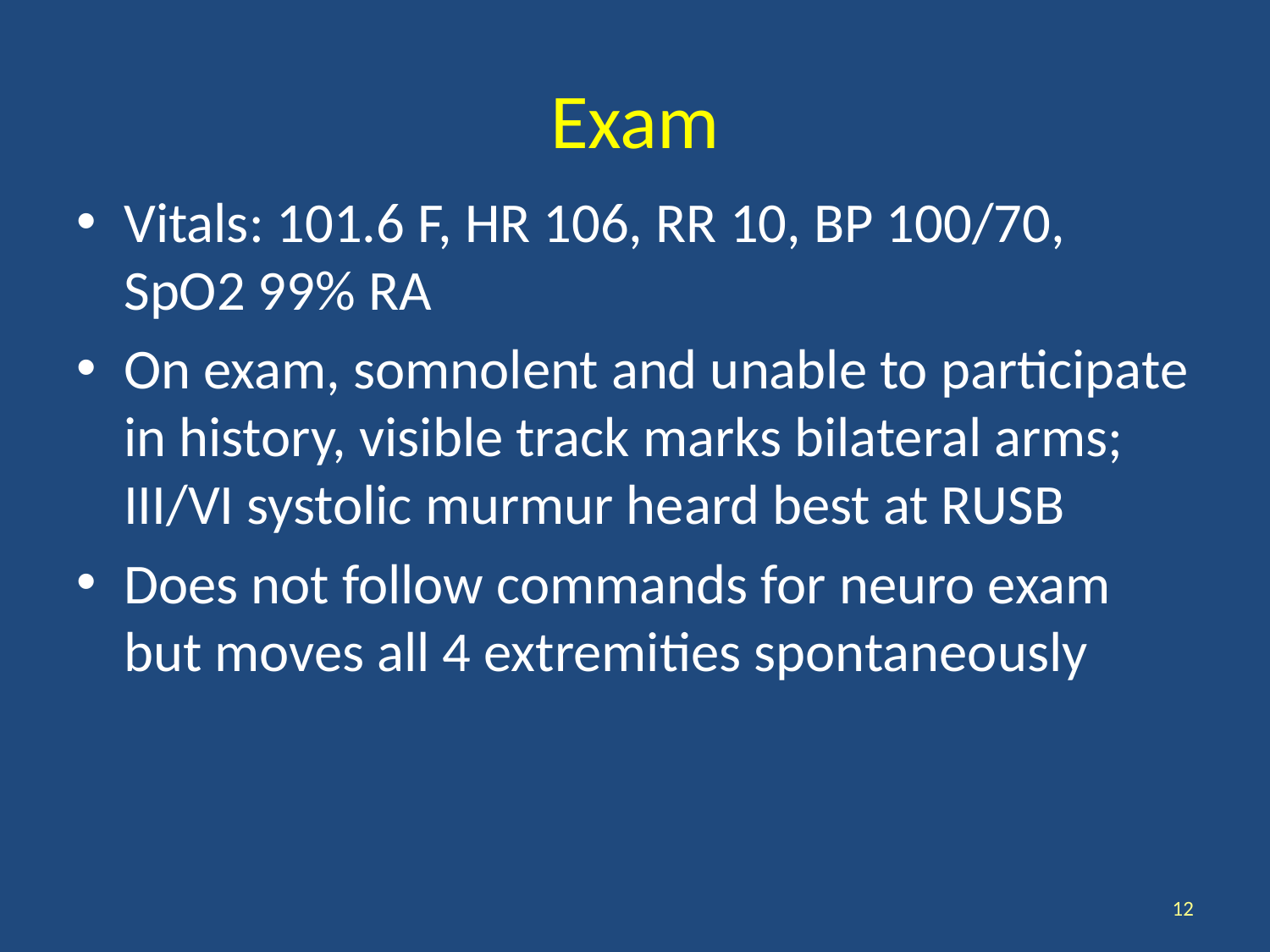

# Exam
Vitals: 101.6 F, HR 106, RR 10, BP 100/70, SpO2 99% RA
On exam, somnolent and unable to participate in history, visible track marks bilateral arms; III/VI systolic murmur heard best at RUSB
Does not follow commands for neuro exam but moves all 4 extremities spontaneously
12

## Slide 13
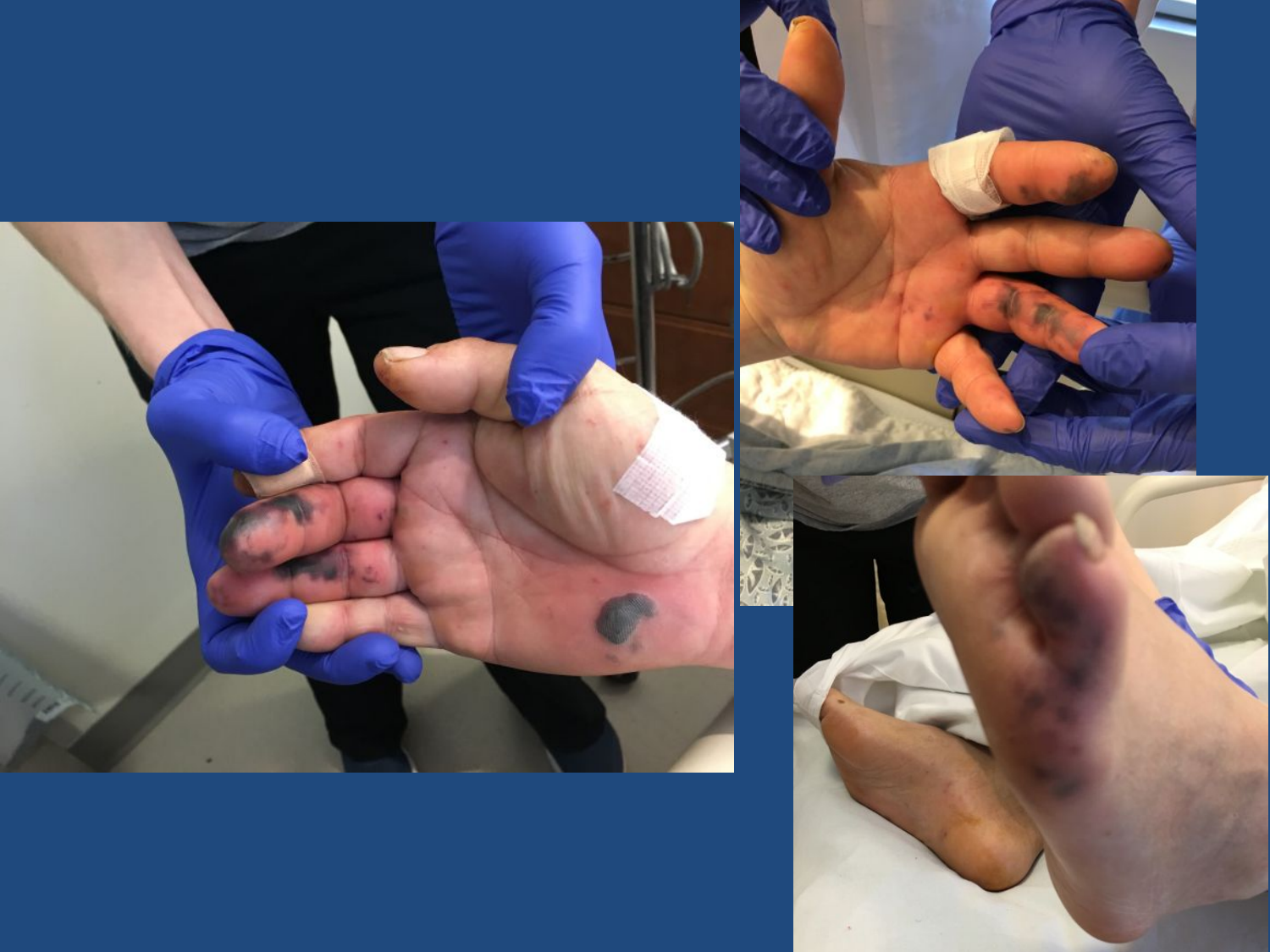

#
13

## Slide 14
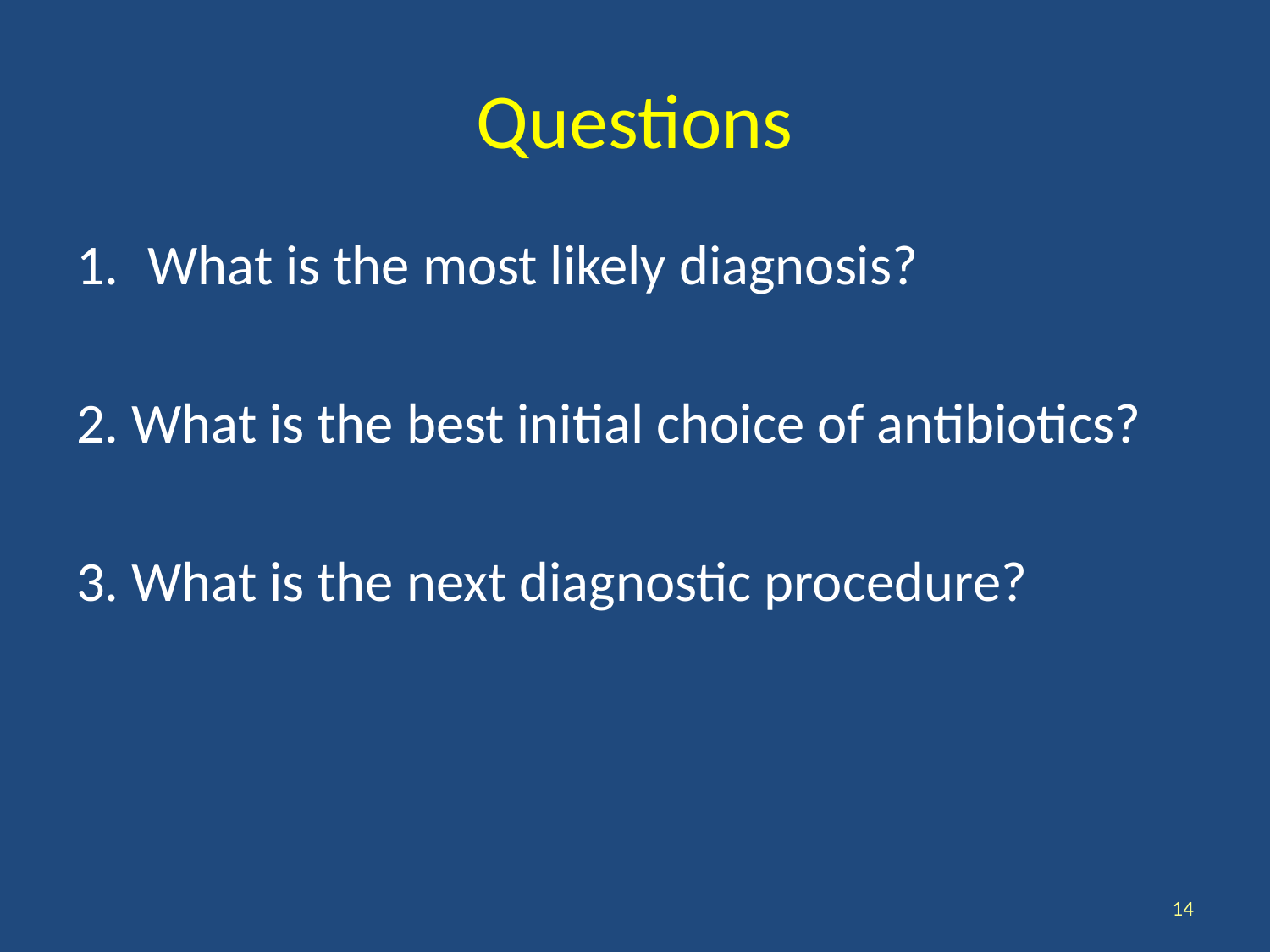

# Questions
What is the most likely diagnosis?
2. What is the best initial choice of antibiotics?
3. What is the next diagnostic procedure?
14

## Slide 15
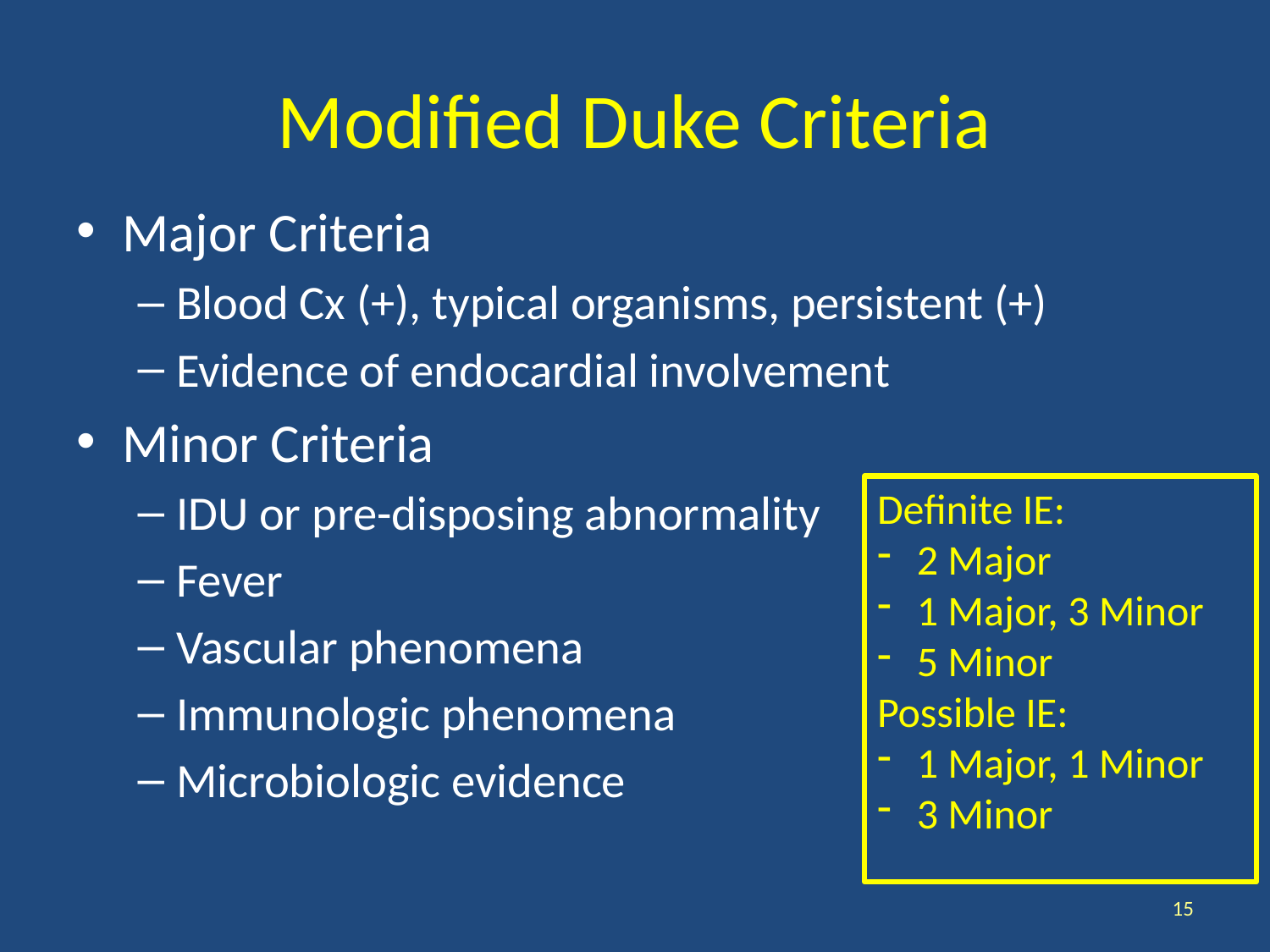

# Modified Duke Criteria
Major Criteria
Blood Cx (+), typical organisms, persistent (+)
Evidence of endocardial involvement
Minor Criteria
IDU or pre-disposing abnormality
Fever
Vascular phenomena
Immunologic phenomena
Microbiologic evidence
Definite IE:
2 Major
1 Major, 3 Minor
5 Minor
Possible IE:
1 Major, 1 Minor
3 Minor
15

## Slide 16
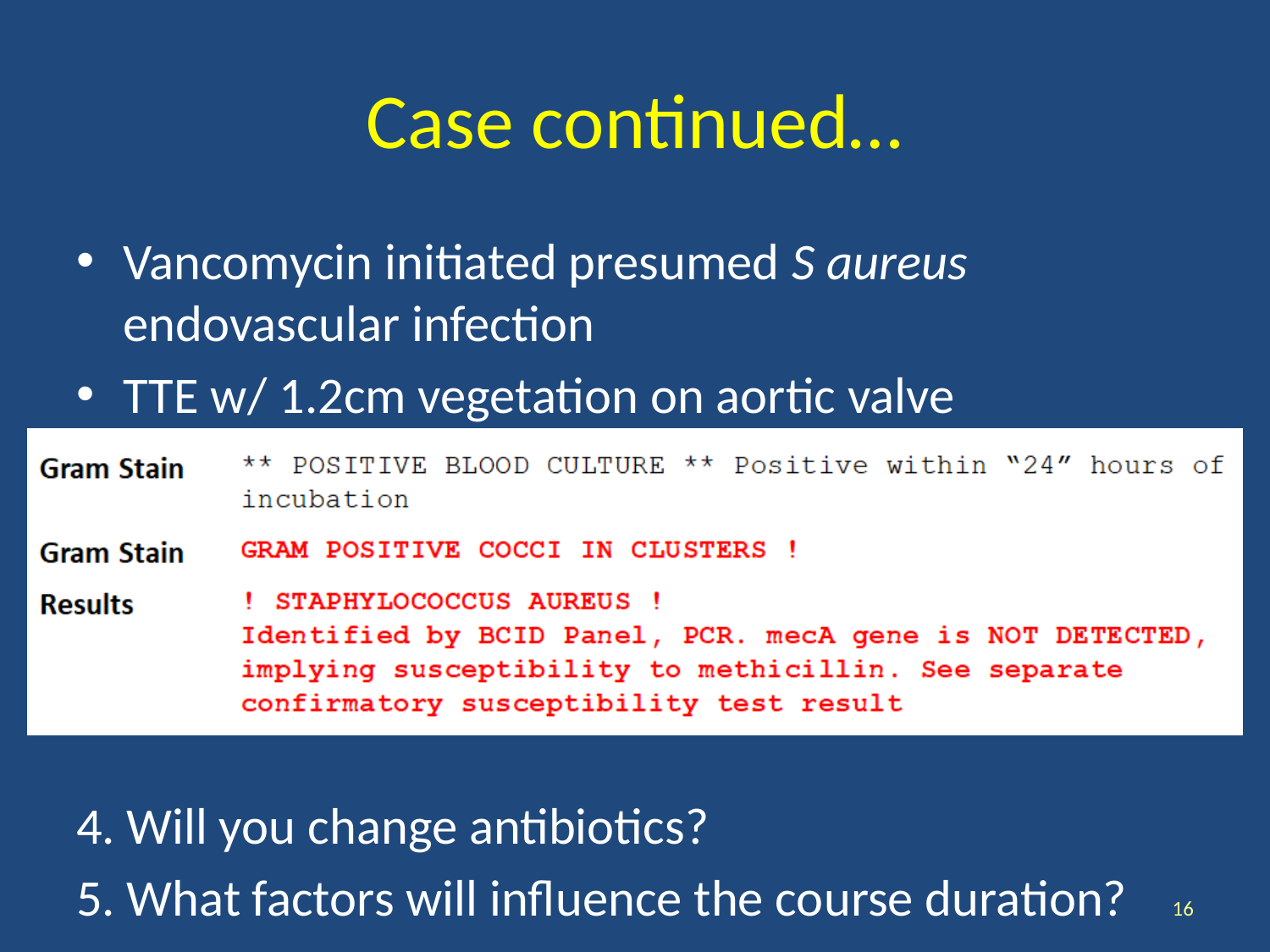

# Case continued…
Vancomycin initiated presumed S aureus endovascular infection
TTE w/ 1.2cm vegetation on aortic valve
You obtain an ID and cardiology consult
Blood cultures:
4. Will you change antibiotics?
5. What factors will influence the course duration?
16

## Slide 17
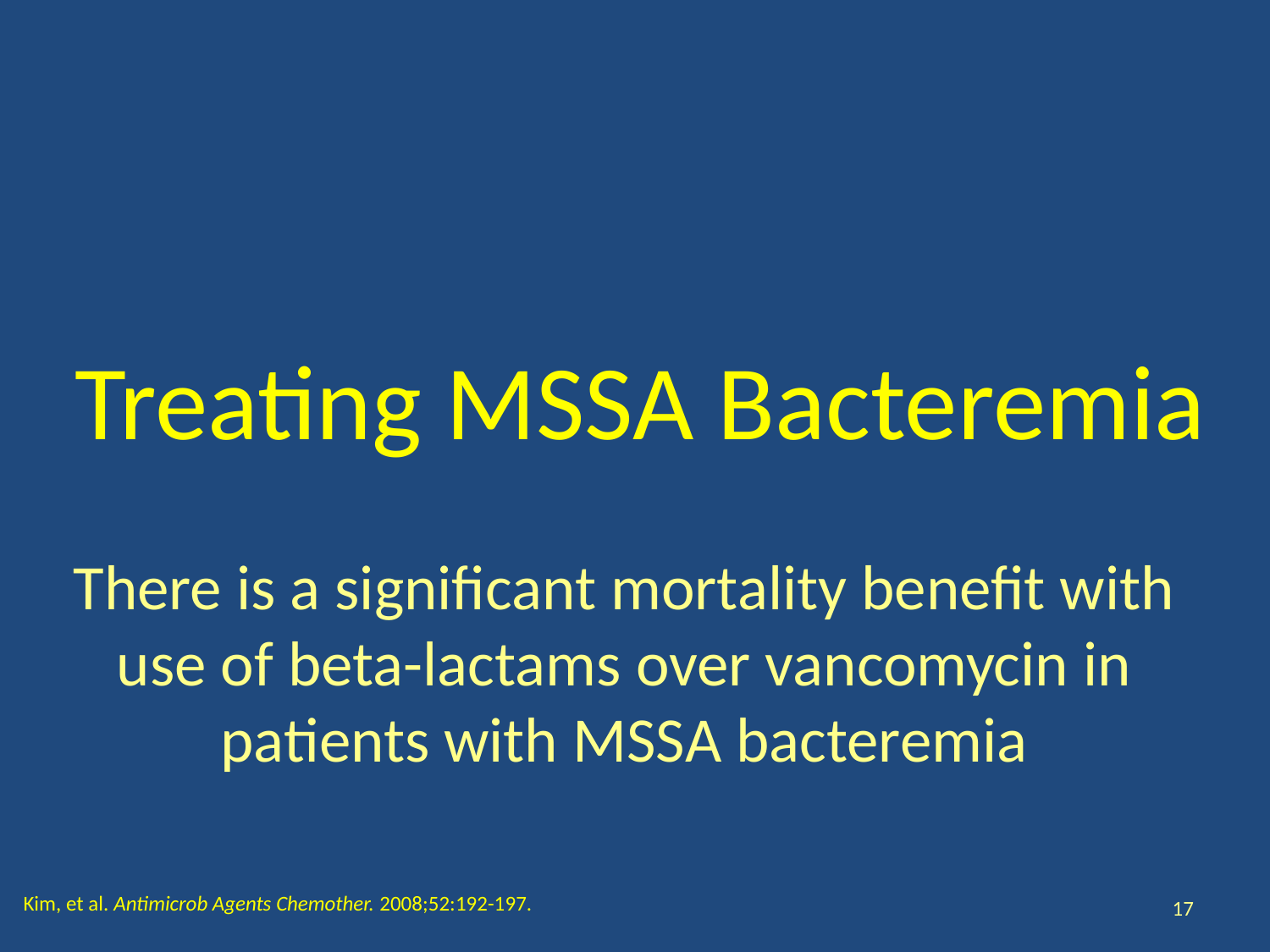

# Treating MSSA Bacteremia
There is a significant mortality benefit with use of beta-lactams over vancomycin in patients with MSSA bacteremia
Kim, et al. Antimicrob Agents Chemother. 2008;52:192-197.
17

## Slide 18
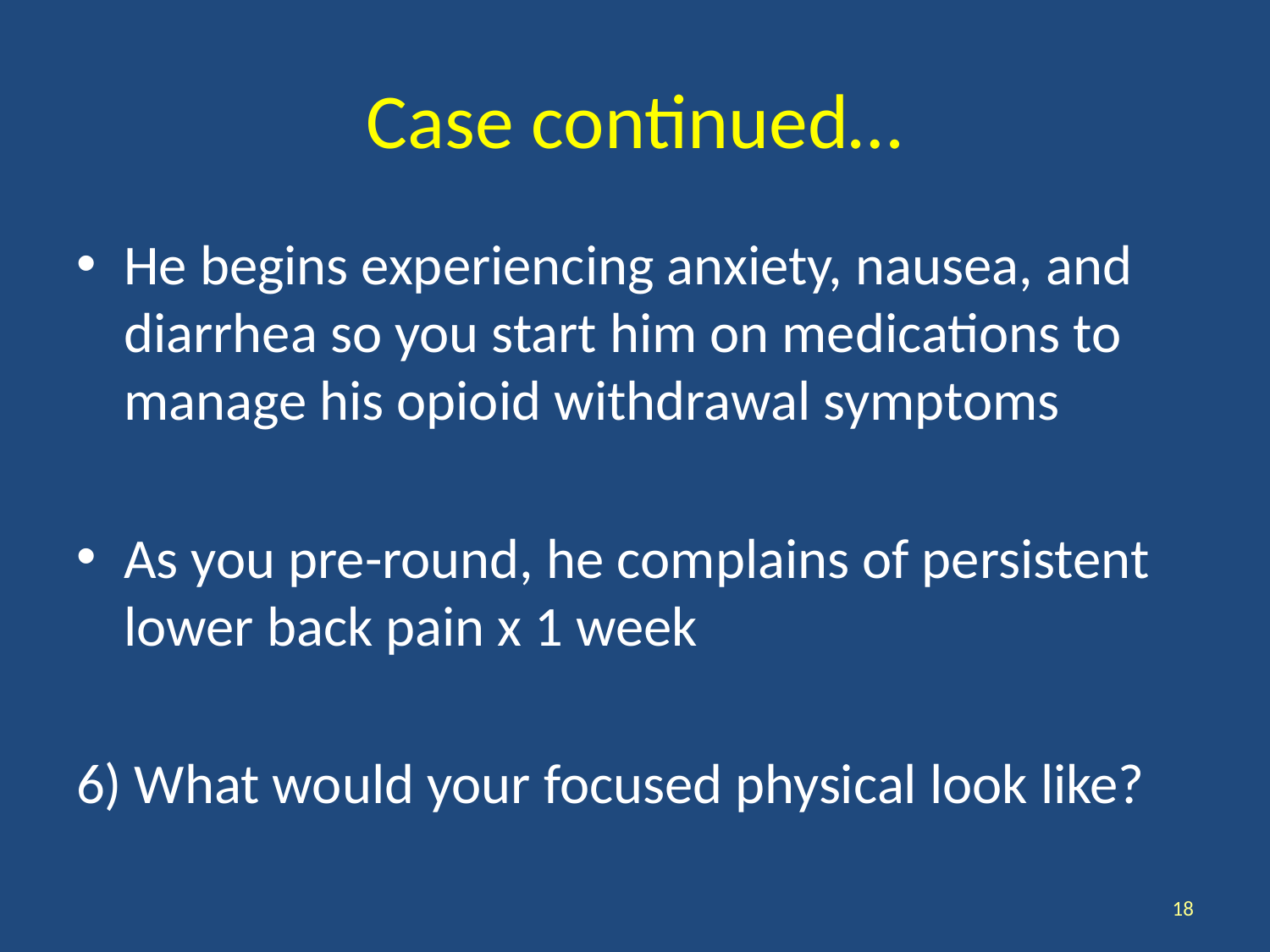

# Case continued…
He begins experiencing anxiety, nausea, and diarrhea so you start him on medications to manage his opioid withdrawal symptoms
As you pre-round, he complains of persistent lower back pain x 1 week
6) What would your focused physical look like?
18

## Slide 19
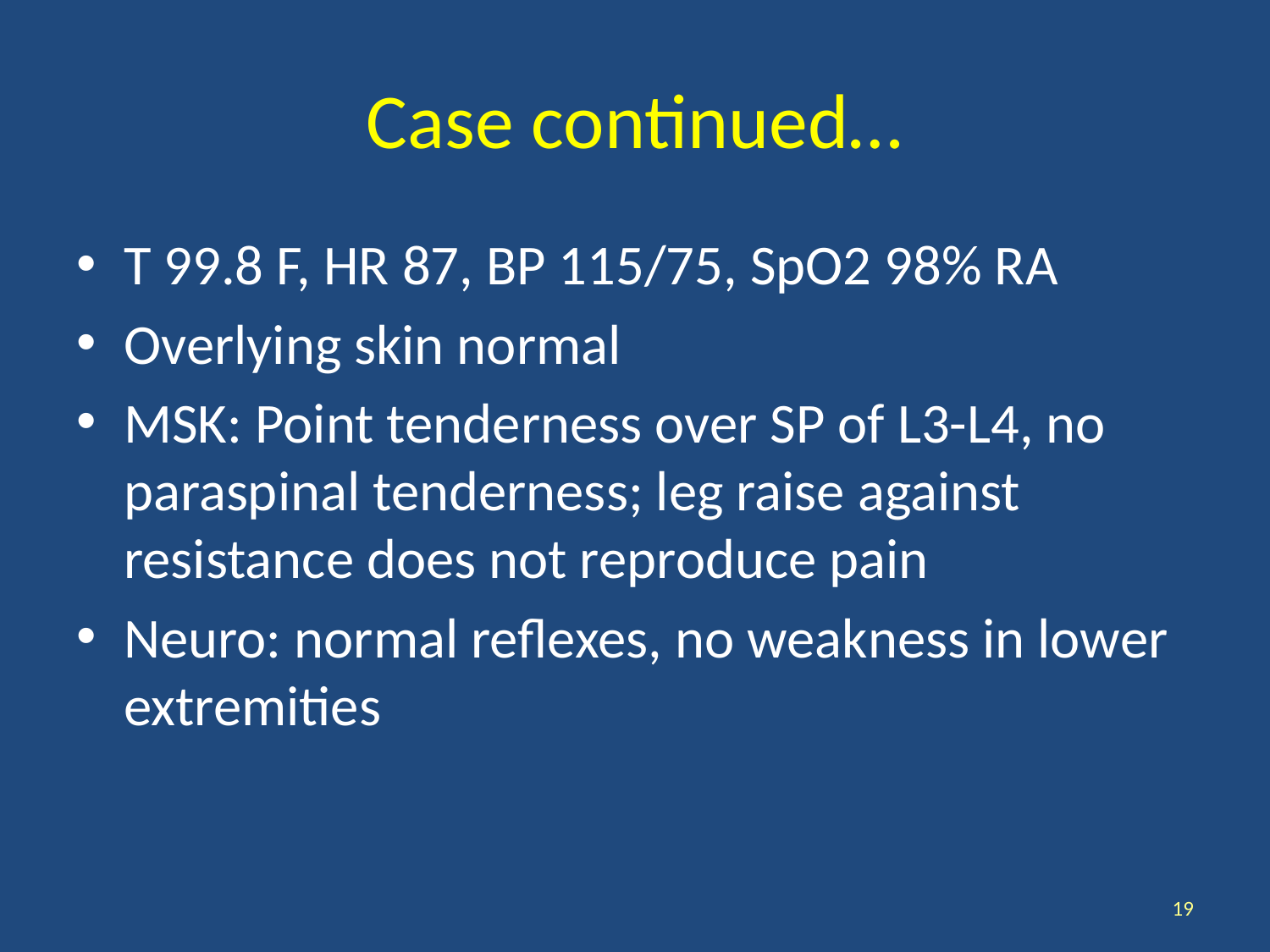

# Case continued…
T 99.8 F, HR 87, BP 115/75, SpO2 98% RA
Overlying skin normal
MSK: Point tenderness over SP of L3-L4, no paraspinal tenderness; leg raise against resistance does not reproduce pain
Neuro: normal reflexes, no weakness in lower extremities
19

## Slide 20
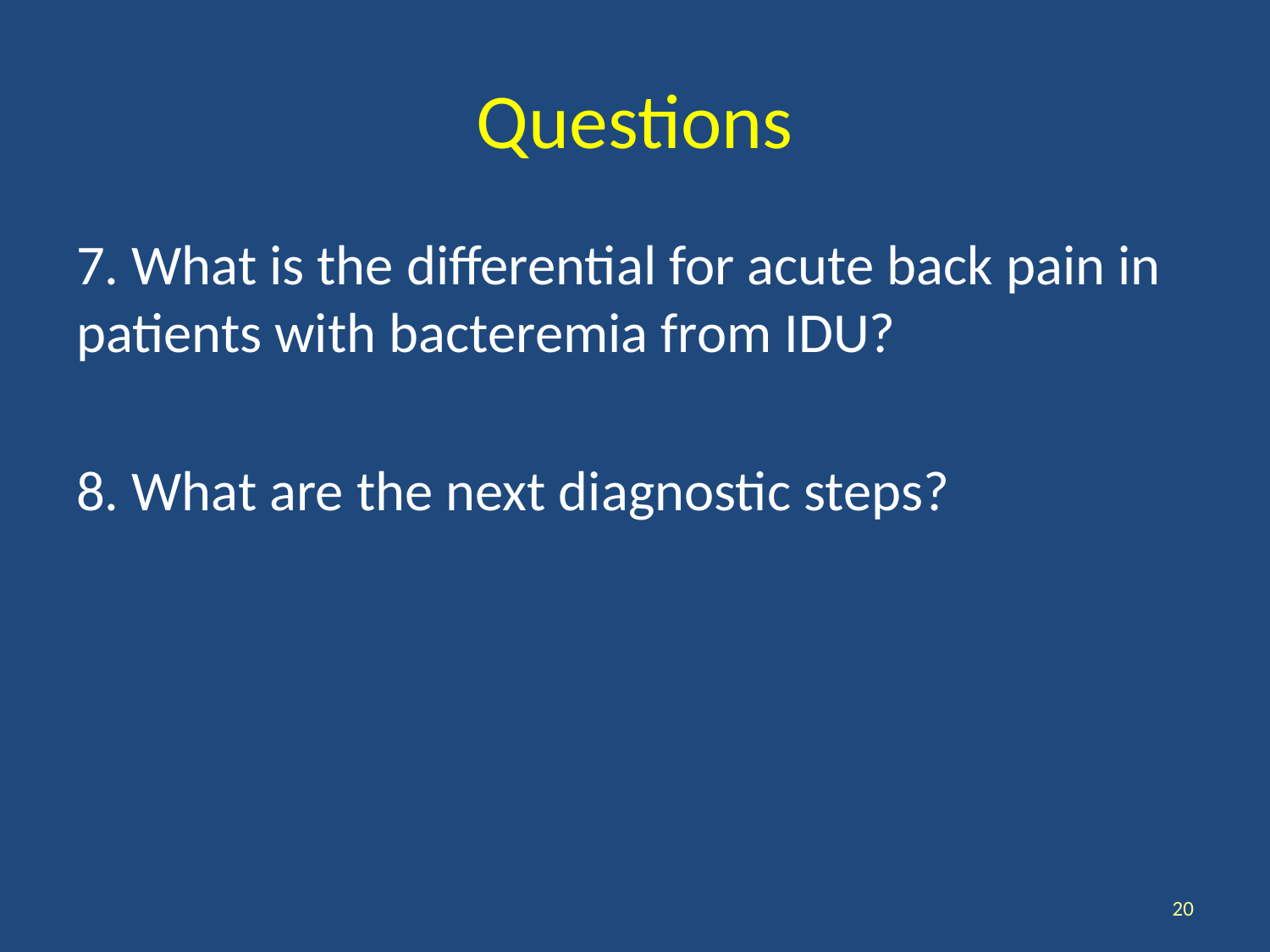

# Questions
7. What is the differential for acute back pain in patients with bacteremia from IDU?
8. What are the next diagnostic steps?
20

## Slide 21
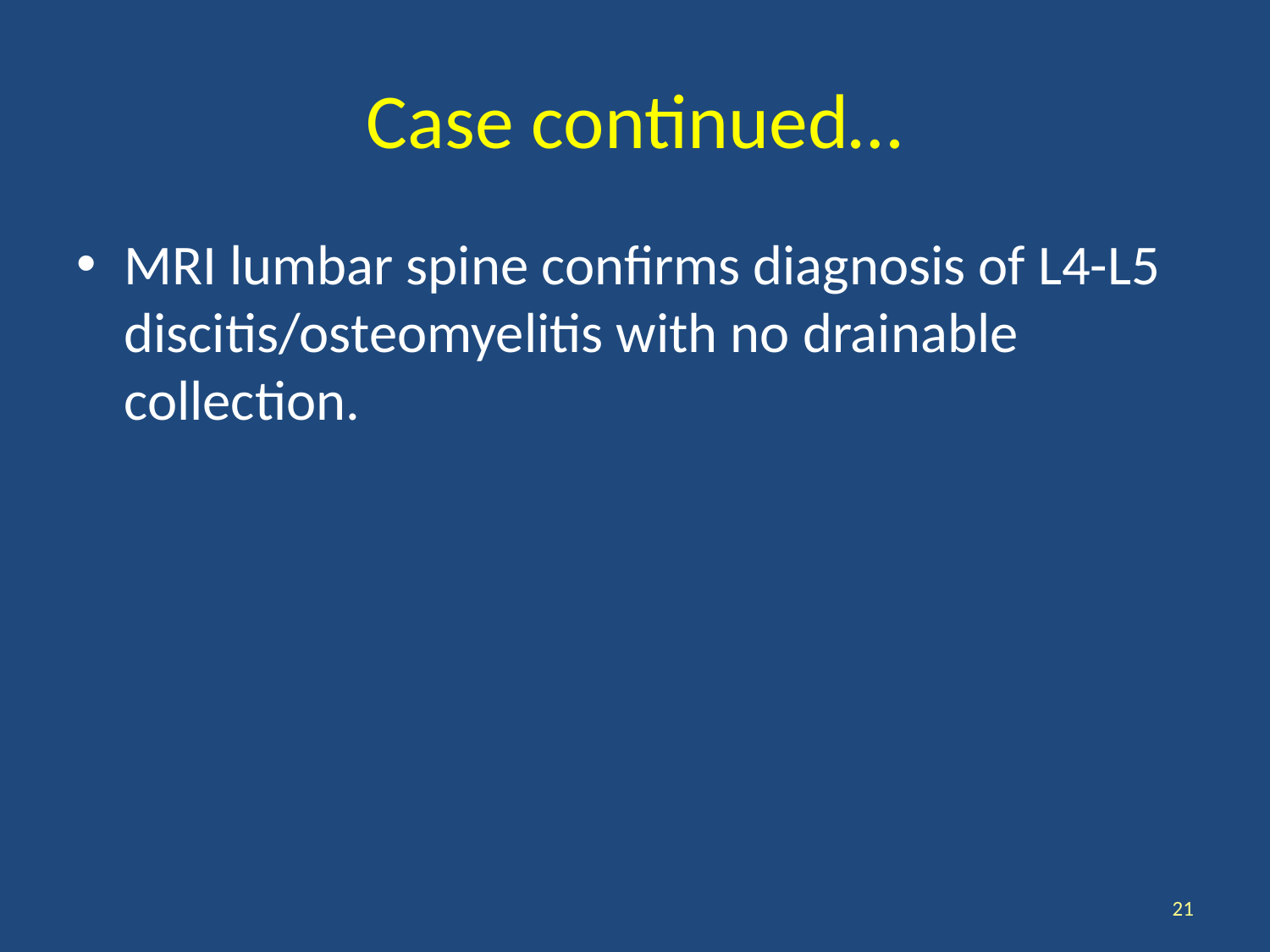

# Case continued…
MRI lumbar spine confirms diagnosis of L4-L5 discitis/osteomyelitis with no drainable collection.
21

## Slide 22
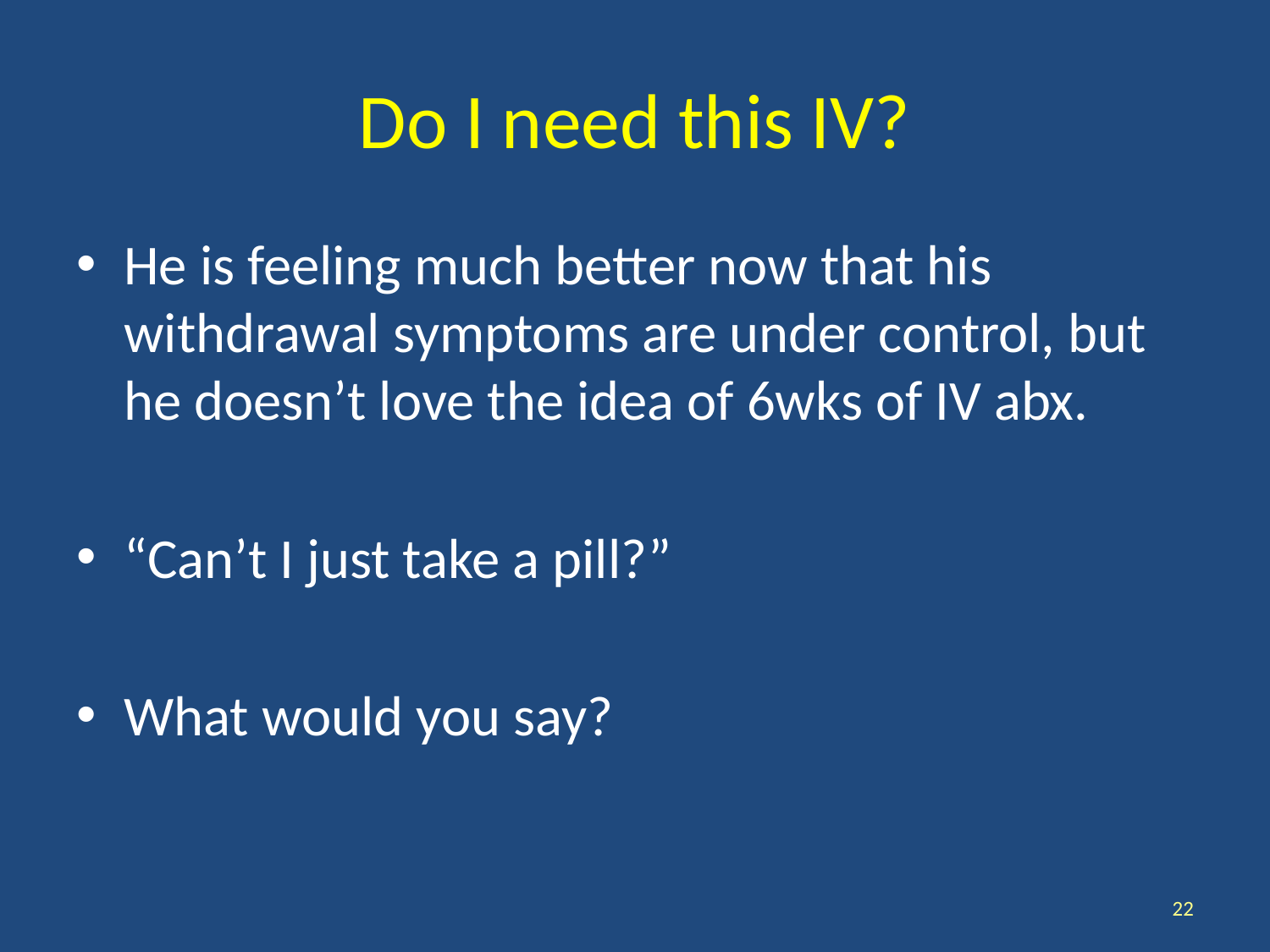

# Do I need this IV?
He is feeling much better now that his withdrawal symptoms are under control, but he doesn’t love the idea of 6wks of IV abx.
“Can’t I just take a pill?”
What would you say?
22

## Slide 23
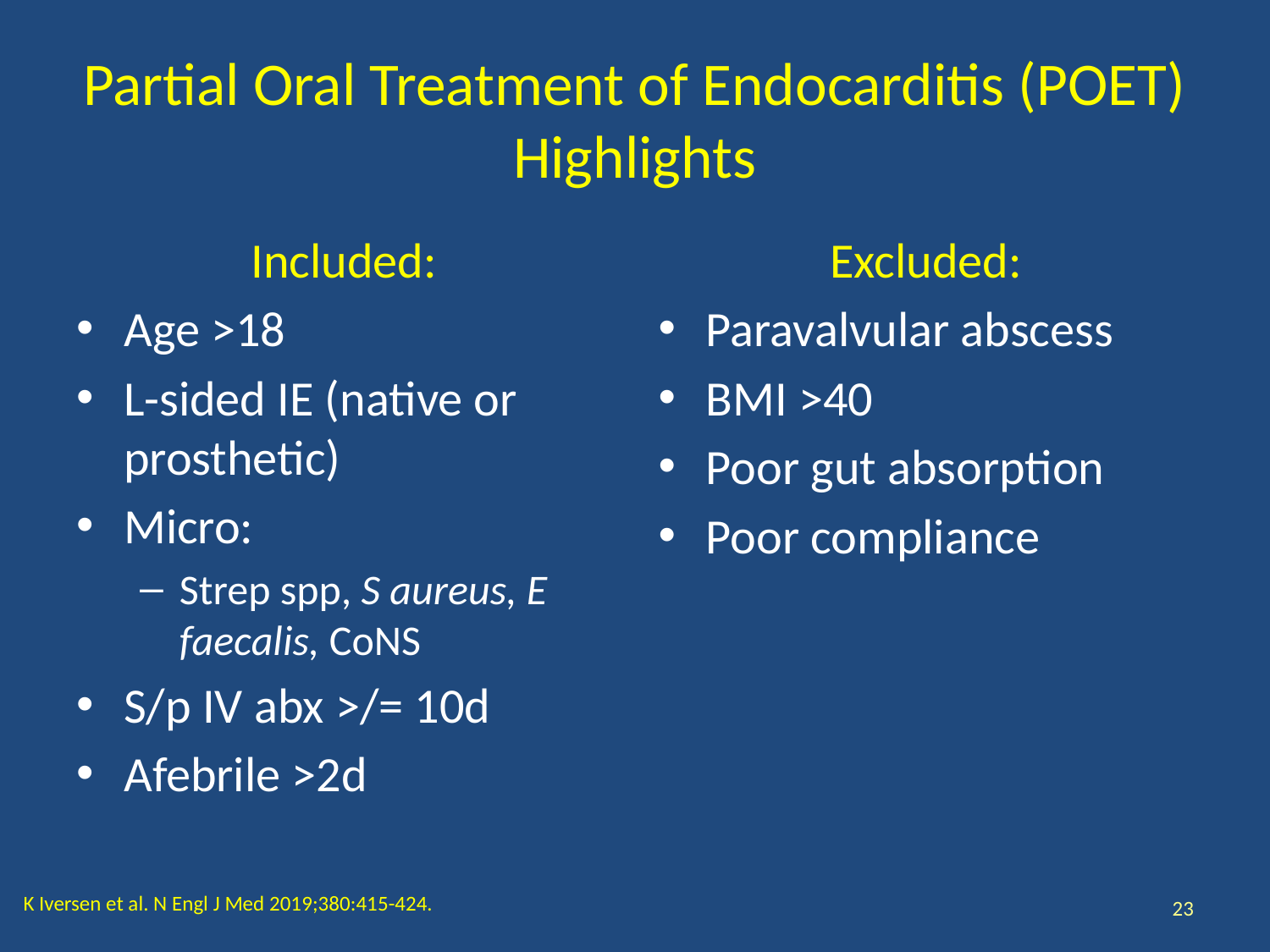

# Partial Oral Treatment of Endocarditis (POET) Highlights
Included:
Age >18
L-sided IE (native or prosthetic)
Micro:
Strep spp, S aureus, E faecalis, CoNS
S/p IV abx >/= 10d
Afebrile >2d
Excluded:
Paravalvular abscess
BMI >40
Poor gut absorption
Poor compliance
K Iversen et al. N Engl J Med 2019;380:415-424.
23

## Slide 24
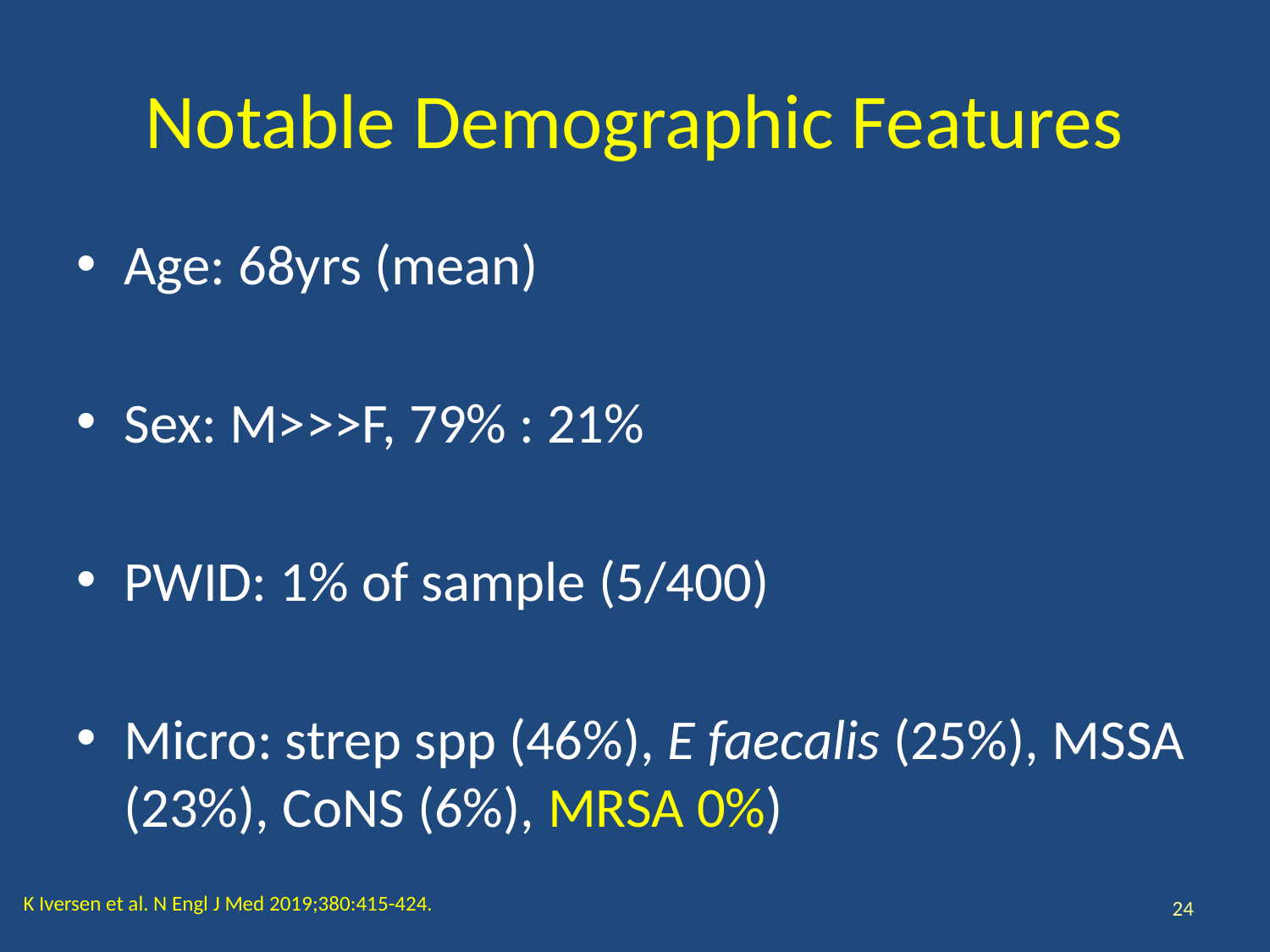

# Notable Demographic Features
Age: 68yrs (mean)
Sex: M>>>F, 79% : 21%
PWID: 1% of sample (5/400)
Micro: strep spp (46%), E faecalis (25%), MSSA (23%), CoNS (6%), MRSA 0%)
K Iversen et al. N Engl J Med 2019;380:415-424.
24

## Slide 25
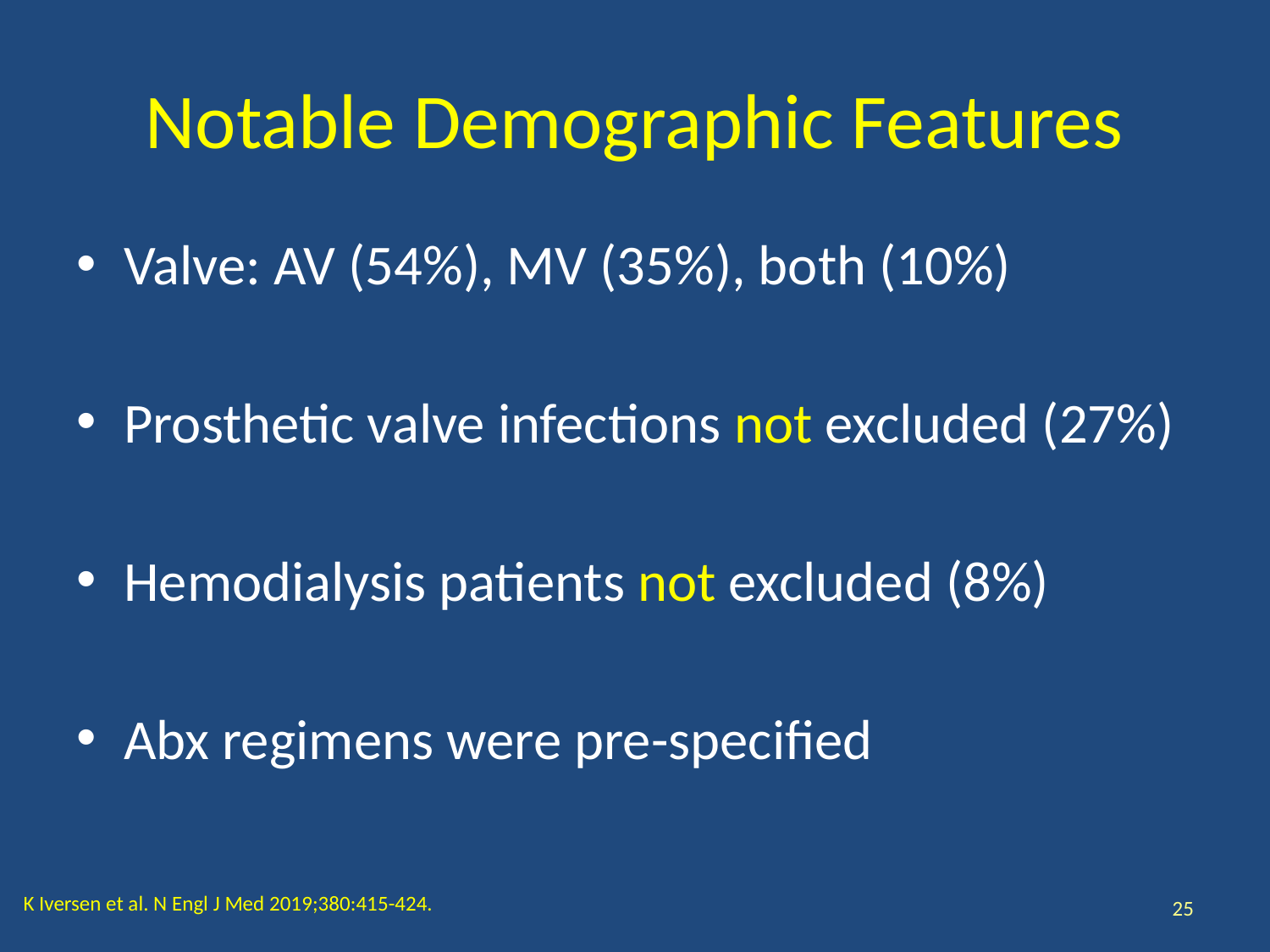

# Notable Demographic Features
Valve: AV (54%), MV (35%), both (10%)
Prosthetic valve infections not excluded (27%)
Hemodialysis patients not excluded (8%)
Abx regimens were pre-specified
K Iversen et al. N Engl J Med 2019;380:415-424.
25

## Slide 26
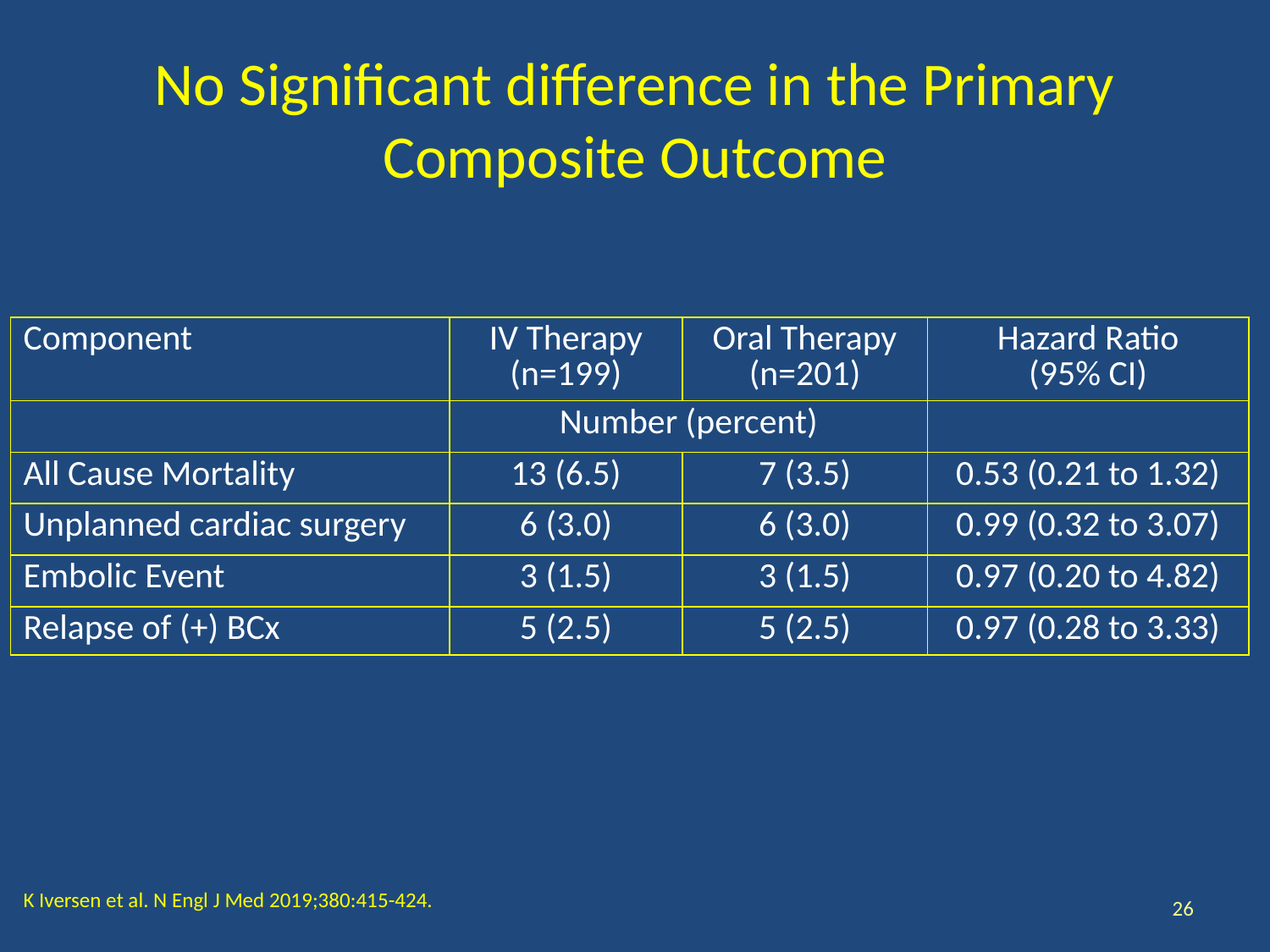

# No Significant difference in the Primary Composite Outcome
| Component | IV Therapy (n=199) | Oral Therapy (n=201) | Hazard Ratio (95% CI) |
| --- | --- | --- | --- |
| | Number (percent) | | |
| All Cause Mortality | 13 (6.5) | 7 (3.5) | 0.53 (0.21 to 1.32) |
| Unplanned cardiac surgery | 6 (3.0) | 6 (3.0) | 0.99 (0.32 to 3.07) |
| Embolic Event | 3 (1.5) | 3 (1.5) | 0.97 (0.20 to 4.82) |
| Relapse of (+) BCx | 5 (2.5) | 5 (2.5) | 0.97 (0.28 to 3.33) |
K Iversen et al. N Engl J Med 2019;380:415-424.
26

## Slide 27
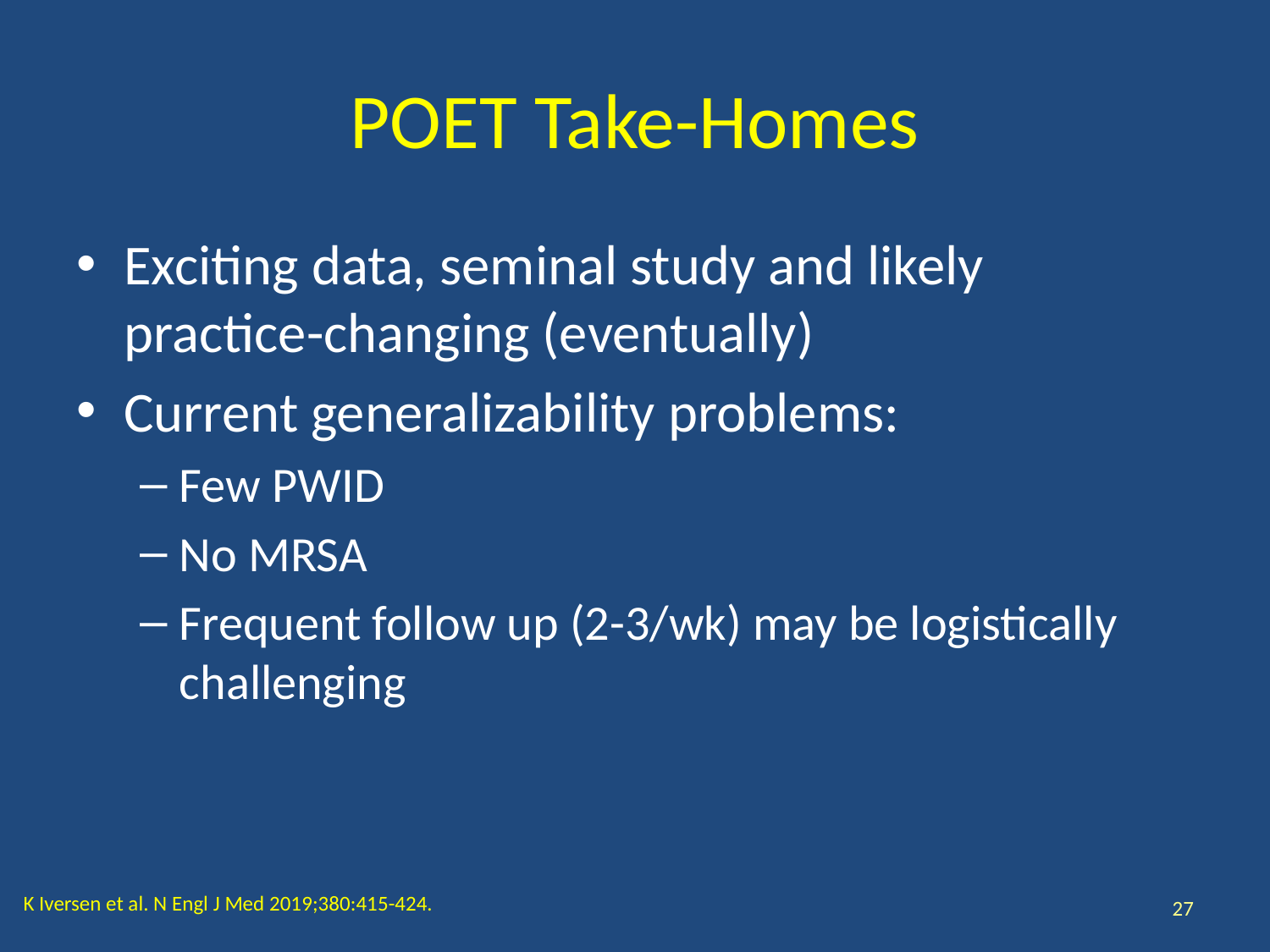

# POET Take-Homes
Exciting data, seminal study and likely practice-changing (eventually)
Current generalizability problems:
Few PWID
No MRSA
Frequent follow up (2-3/wk) may be logistically challenging
K Iversen et al. N Engl J Med 2019;380:415-424.
27

## Slide 28
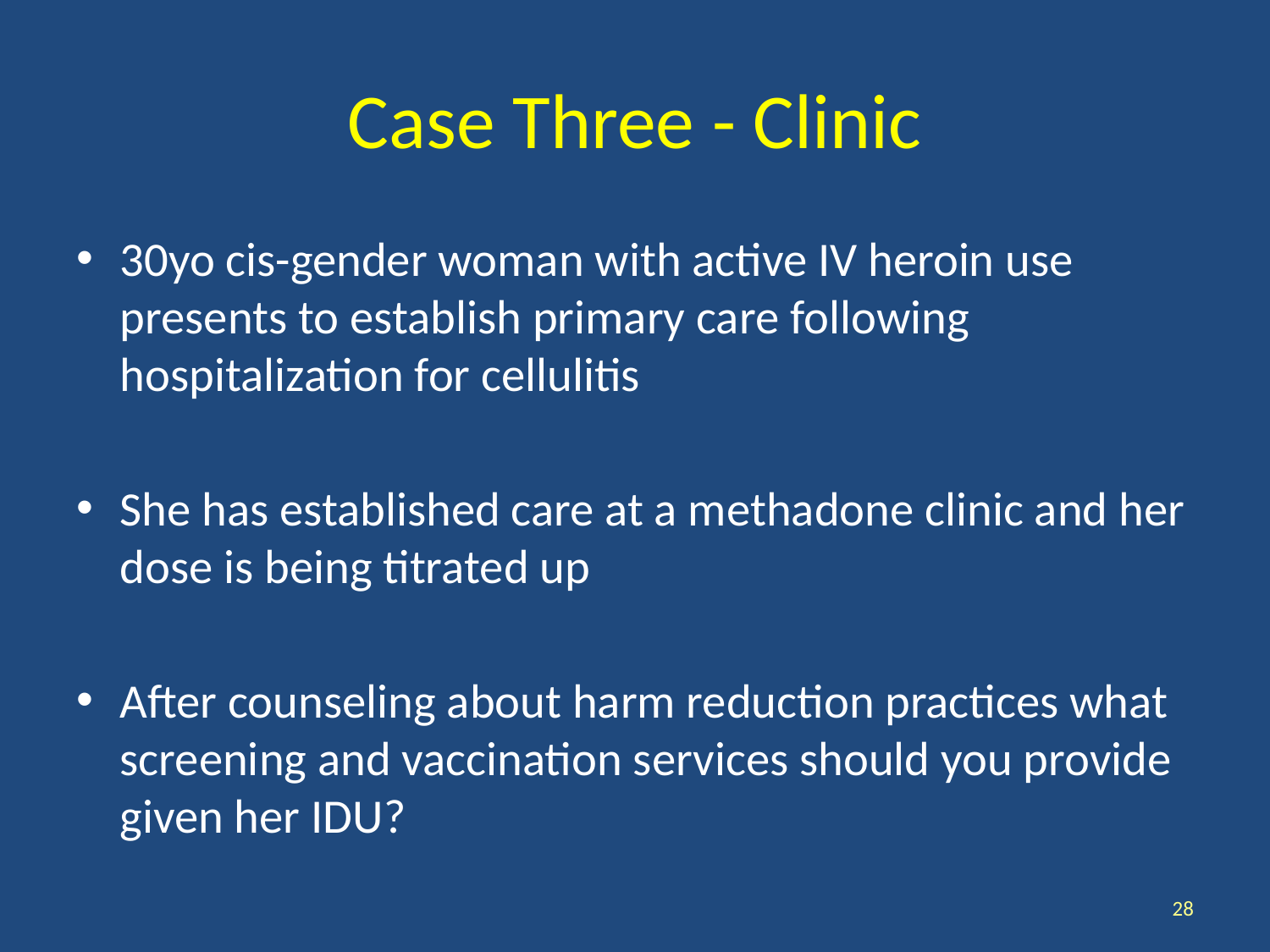

# Case Three - Clinic
30yo cis-gender woman with active IV heroin use presents to establish primary care following hospitalization for cellulitis
She has established care at a methadone clinic and her dose is being titrated up
After counseling about harm reduction practices what screening and vaccination services should you provide given her IDU?
28

## Slide 29
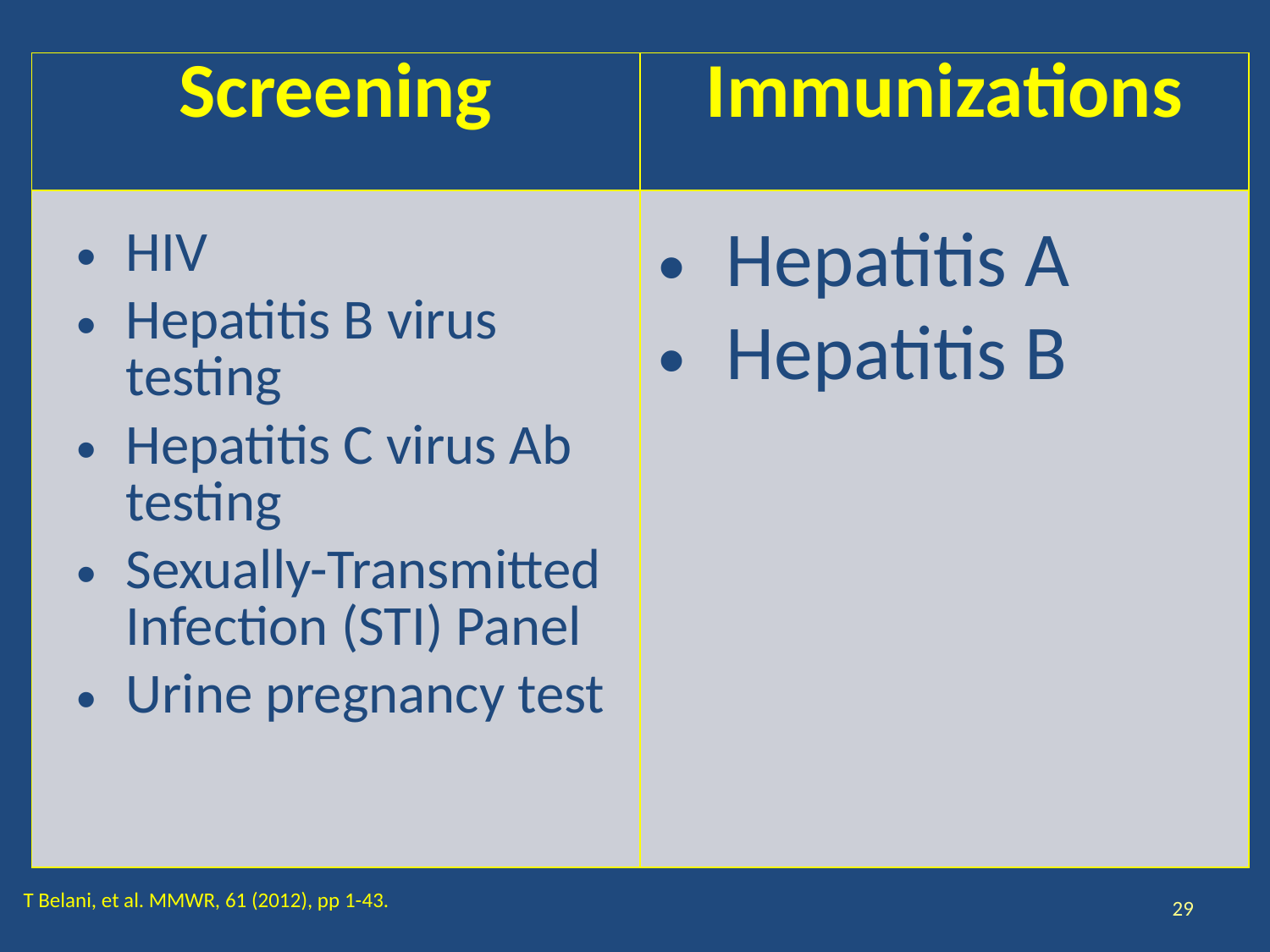

| Screening | Immunizations |
| --- | --- |
| | |
HIV
Hepatitis B virus testing
Hepatitis C virus Ab testing
Sexually-Transmitted Infection (STI) Panel
Urine pregnancy test
Hepatitis A
Hepatitis B
T Belani, et al. MMWR, 61 (2012), pp 1-43.
29

## Slide 30
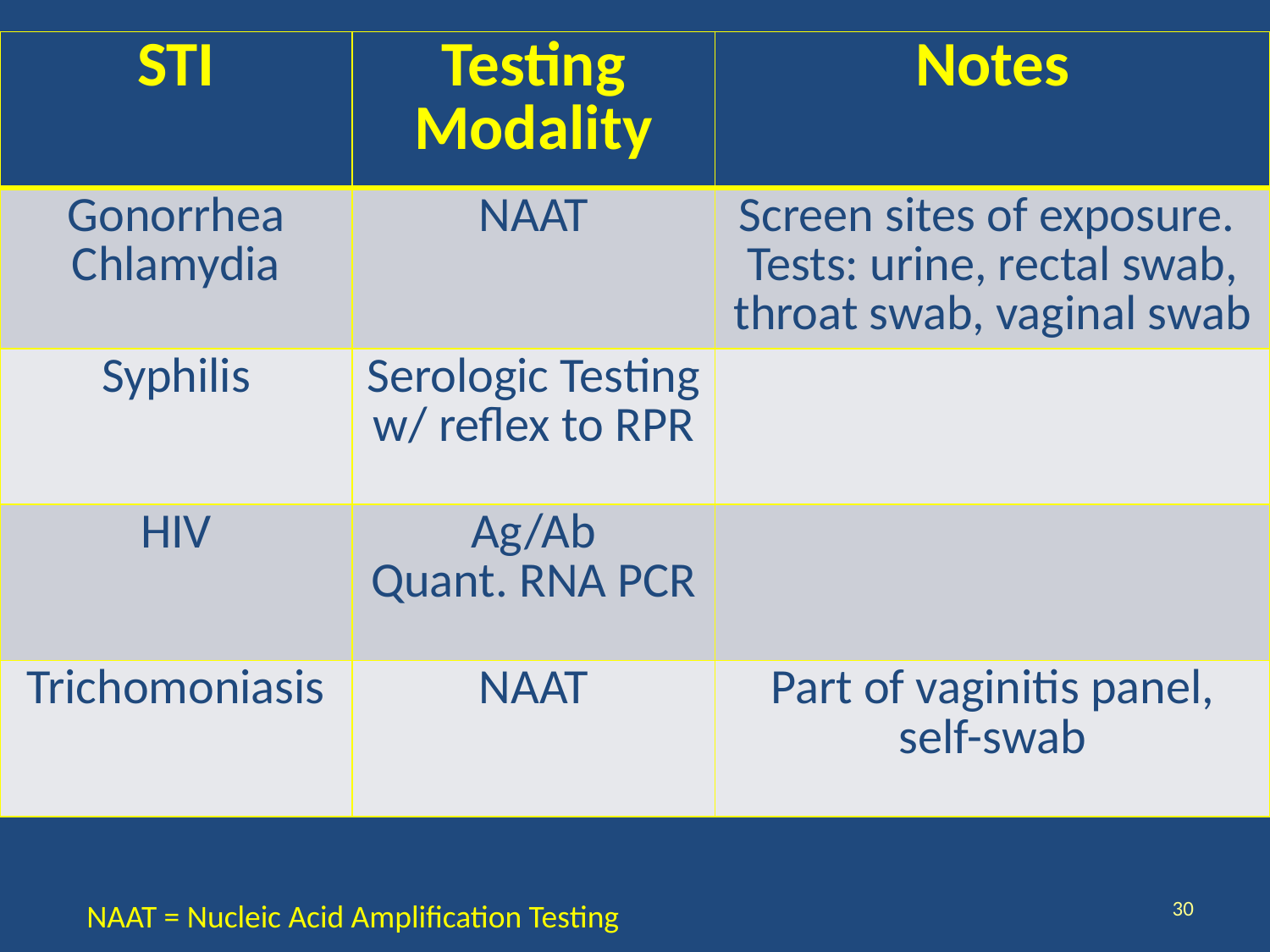

| STI | Testing Modality | Notes |
| --- | --- | --- |
| Gonorrhea Chlamydia | NAAT | Screen sites of exposure. Tests: urine, rectal swab, throat swab, vaginal swab |
| Syphilis | Serologic Testing w/ reflex to RPR | |
| HIV | Ag/Ab Quant. RNA PCR | |
| Trichomoniasis | NAAT | Part of vaginitis panel, self-swab |
#
30
NAAT = Nucleic Acid Amplification Testing

## Slide 31
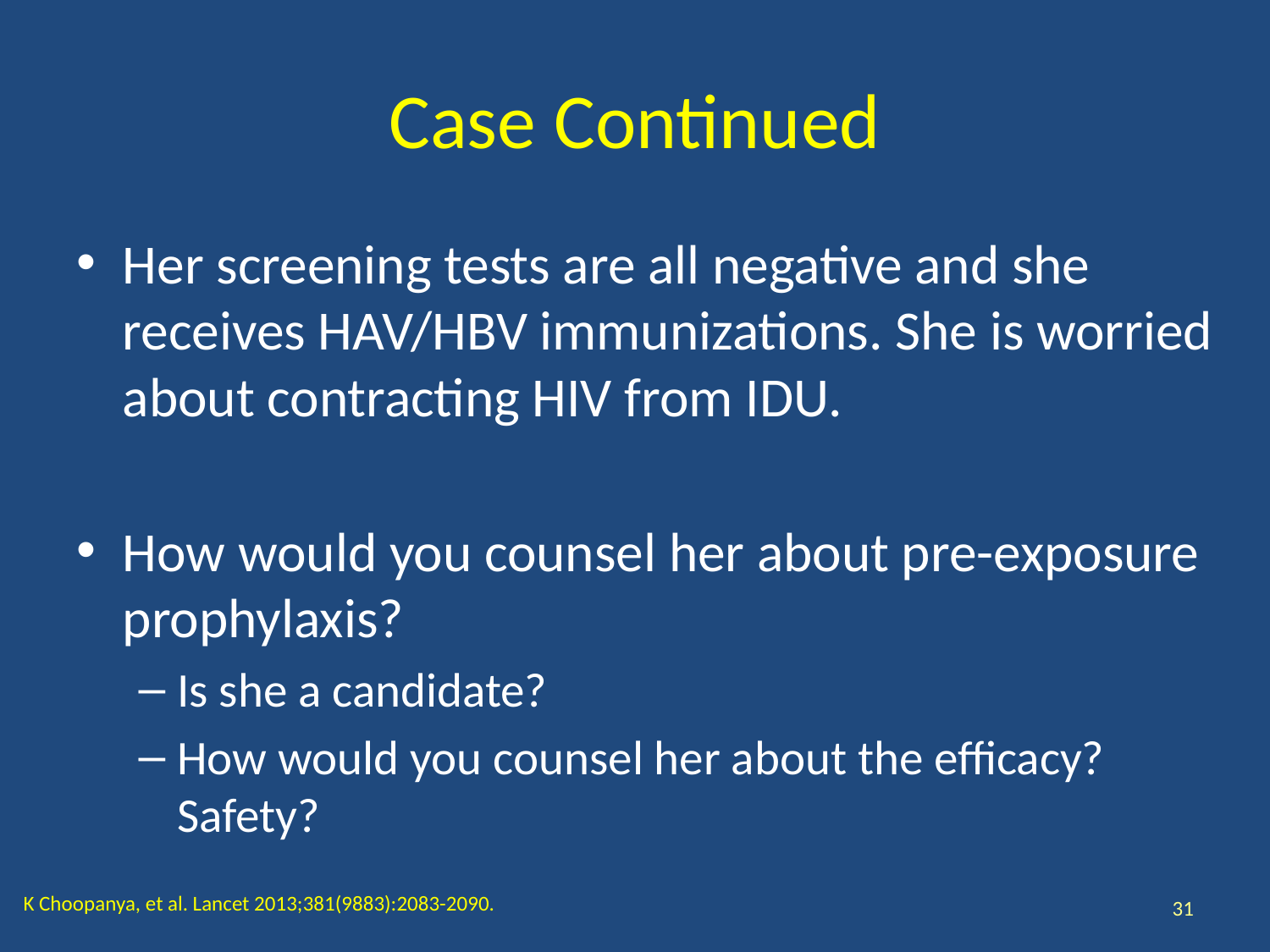

# Case Continued
Her screening tests are all negative and she receives HAV/HBV immunizations. She is worried about contracting HIV from IDU.
How would you counsel her about pre-exposure prophylaxis?
Is she a candidate?
How would you counsel her about the efficacy? Safety?
K Choopanya, et al. Lancet 2013;381(9883):2083-2090.
31

## Slide 32
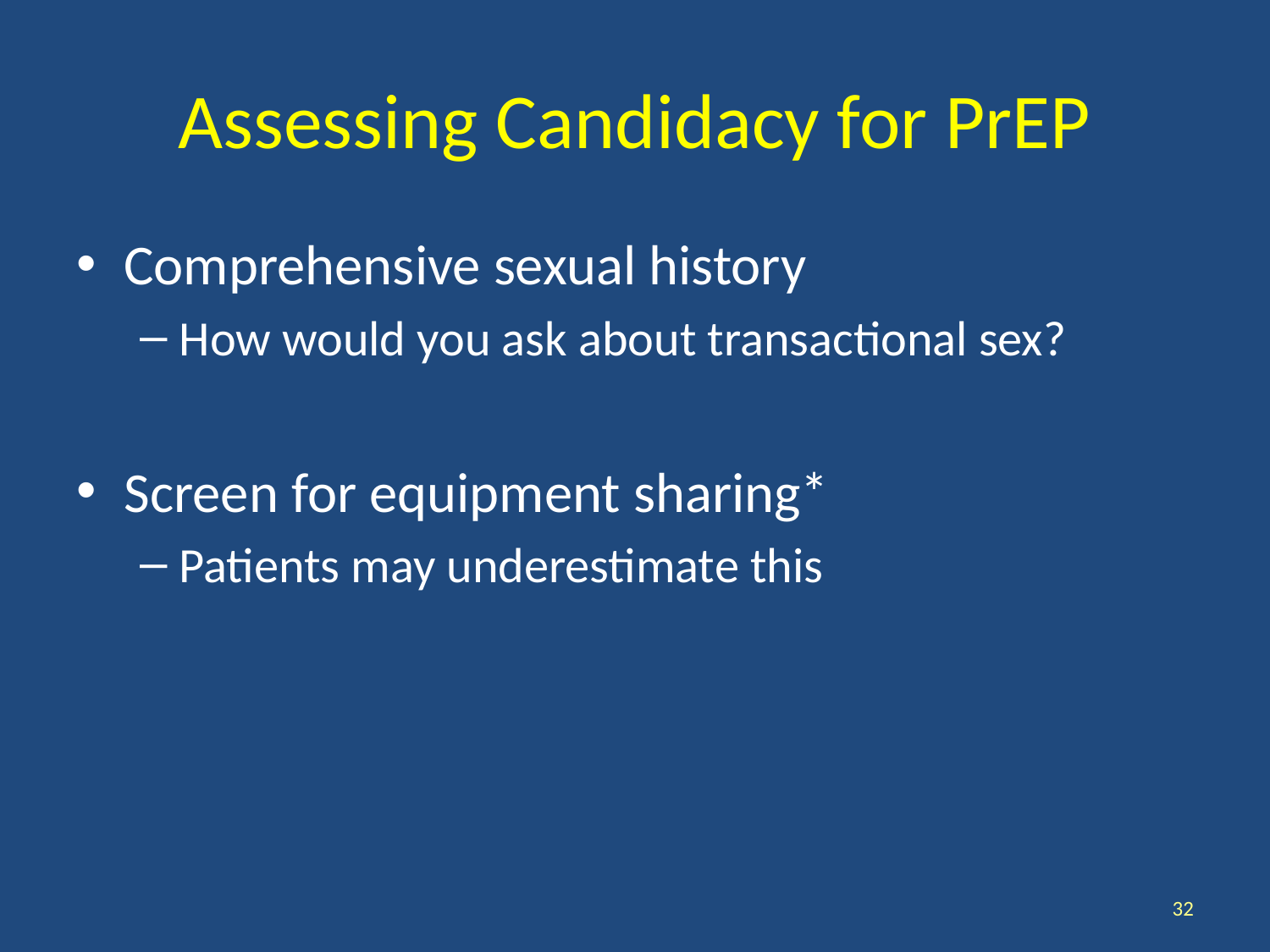

# Assessing Candidacy for PrEP
Comprehensive sexual history
How would you ask about transactional sex?
Screen for equipment sharing*
Patients may underestimate this
32

## Slide 33
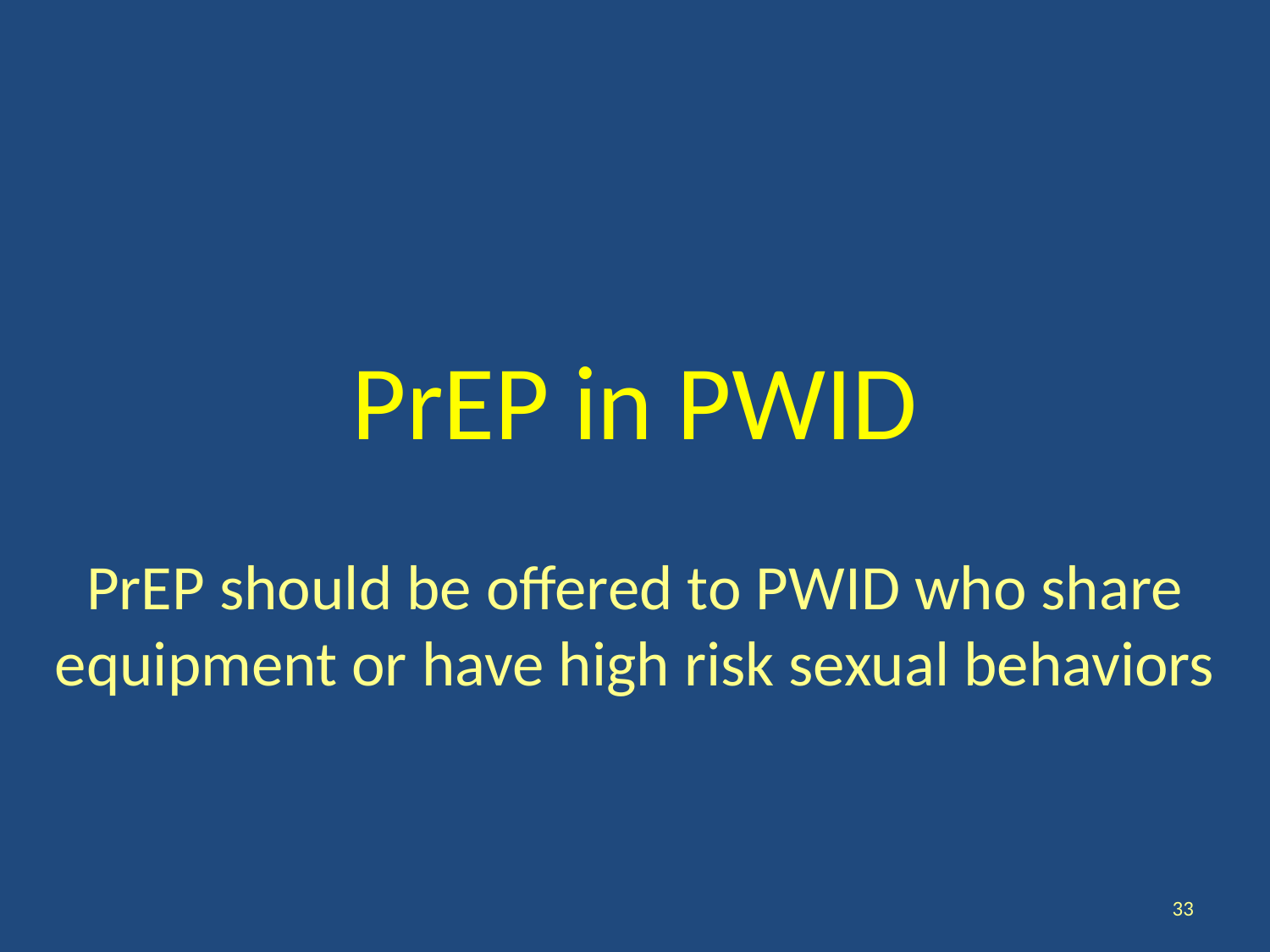

# PrEP in PWID
PrEP should be offered to PWID who share equipment or have high risk sexual behaviors
33

## Slide 34
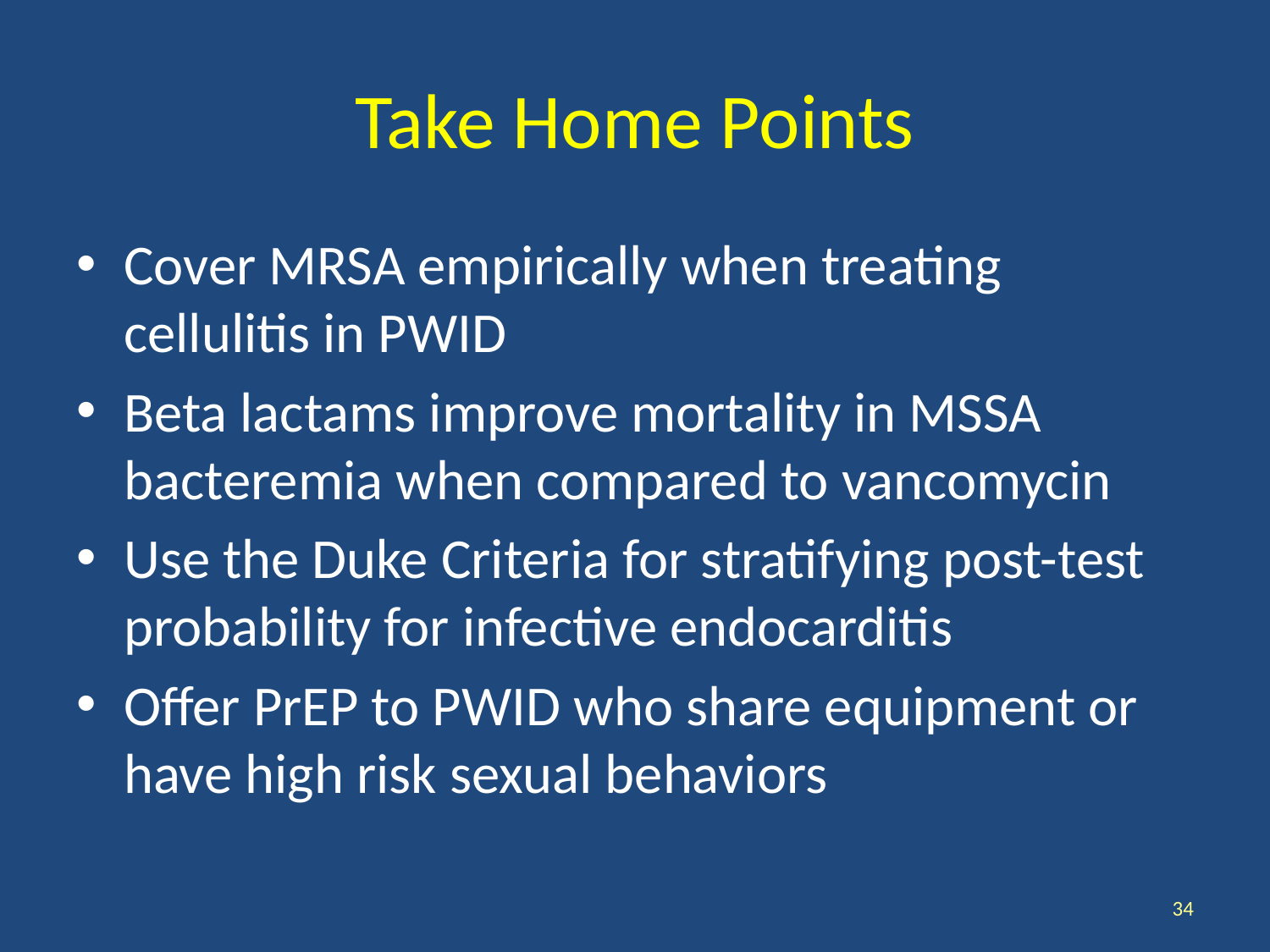

# Take Home Points
Cover MRSA empirically when treating cellulitis in PWID
Beta lactams improve mortality in MSSA bacteremia when compared to vancomycin
Use the Duke Criteria for stratifying post-test probability for infective endocarditis
Offer PrEP to PWID who share equipment or have high risk sexual behaviors
34

## Slide 35
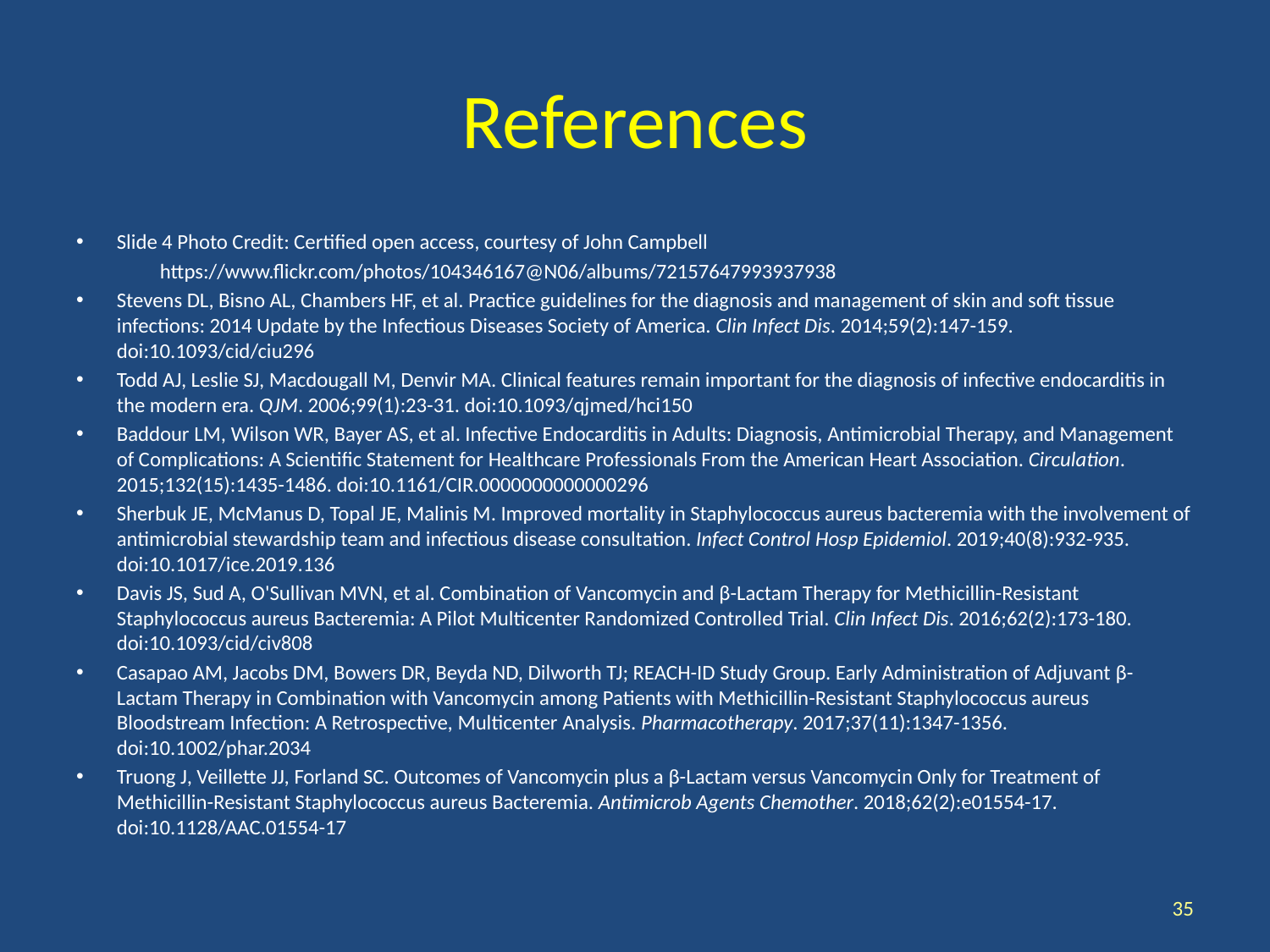

# References
Slide 4 Photo Credit: Certified open access, courtesy of John Campbell
	https://www.flickr.com/photos/104346167@N06/albums/72157647993937938
Stevens DL, Bisno AL, Chambers HF, et al. Practice guidelines for the diagnosis and management of skin and soft tissue infections: 2014 Update by the Infectious Diseases Society of America. Clin Infect Dis. 2014;59(2):147-159. doi:10.1093/cid/ciu296
Todd AJ, Leslie SJ, Macdougall M, Denvir MA. Clinical features remain important for the diagnosis of infective endocarditis in the modern era. QJM. 2006;99(1):23-31. doi:10.1093/qjmed/hci150
Baddour LM, Wilson WR, Bayer AS, et al. Infective Endocarditis in Adults: Diagnosis, Antimicrobial Therapy, and Management of Complications: A Scientific Statement for Healthcare Professionals From the American Heart Association. Circulation. 2015;132(15):1435-1486. doi:10.1161/CIR.0000000000000296
Sherbuk JE, McManus D, Topal JE, Malinis M. Improved mortality in Staphylococcus aureus bacteremia with the involvement of antimicrobial stewardship team and infectious disease consultation. Infect Control Hosp Epidemiol. 2019;40(8):932-935. doi:10.1017/ice.2019.136
Davis JS, Sud A, O'Sullivan MVN, et al. Combination of Vancomycin and β-Lactam Therapy for Methicillin-Resistant Staphylococcus aureus Bacteremia: A Pilot Multicenter Randomized Controlled Trial. Clin Infect Dis. 2016;62(2):173-180. doi:10.1093/cid/civ808
Casapao AM, Jacobs DM, Bowers DR, Beyda ND, Dilworth TJ; REACH-ID Study Group. Early Administration of Adjuvant β-Lactam Therapy in Combination with Vancomycin among Patients with Methicillin-Resistant Staphylococcus aureus Bloodstream Infection: A Retrospective, Multicenter Analysis. Pharmacotherapy. 2017;37(11):1347-1356. doi:10.1002/phar.2034
Truong J, Veillette JJ, Forland SC. Outcomes of Vancomycin plus a β-Lactam versus Vancomycin Only for Treatment of Methicillin-Resistant Staphylococcus aureus Bacteremia. Antimicrob Agents Chemother. 2018;62(2):e01554-17. doi:10.1128/AAC.01554-17
35

## Slide 36
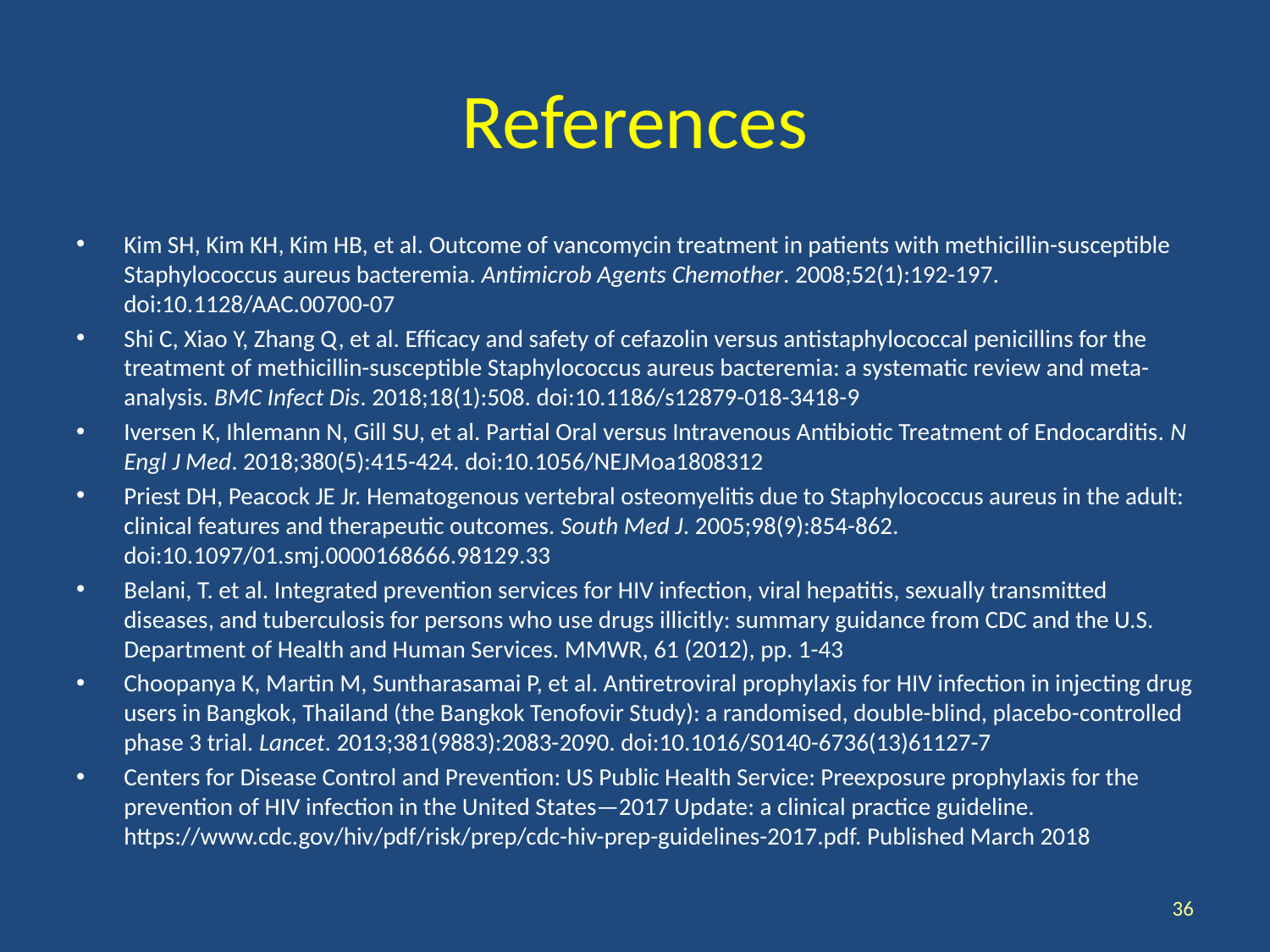

# References
Kim SH, Kim KH, Kim HB, et al. Outcome of vancomycin treatment in patients with methicillin-susceptible Staphylococcus aureus bacteremia. Antimicrob Agents Chemother. 2008;52(1):192-197. doi:10.1128/AAC.00700-07
Shi C, Xiao Y, Zhang Q, et al. Efficacy and safety of cefazolin versus antistaphylococcal penicillins for the treatment of methicillin-susceptible Staphylococcus aureus bacteremia: a systematic review and meta-analysis. BMC Infect Dis. 2018;18(1):508. doi:10.1186/s12879-018-3418-9
Iversen K, Ihlemann N, Gill SU, et al. Partial Oral versus Intravenous Antibiotic Treatment of Endocarditis. N Engl J Med. 2018;380(5):415-424. doi:10.1056/NEJMoa1808312
Priest DH, Peacock JE Jr. Hematogenous vertebral osteomyelitis due to Staphylococcus aureus in the adult: clinical features and therapeutic outcomes. South Med J. 2005;98(9):854-862. doi:10.1097/01.smj.0000168666.98129.33
Belani, T. et al. Integrated prevention services for HIV infection, viral hepatitis, sexually transmitted diseases, and tuberculosis for persons who use drugs illicitly: summary guidance from CDC and the U.S. Department of Health and Human Services. MMWR, 61 (2012), pp. 1-43
Choopanya K, Martin M, Suntharasamai P, et al. Antiretroviral prophylaxis for HIV infection in injecting drug users in Bangkok, Thailand (the Bangkok Tenofovir Study): a randomised, double-blind, placebo-controlled phase 3 trial. Lancet. 2013;381(9883):2083-2090. doi:10.1016/S0140-6736(13)61127-7
Centers for Disease Control and Prevention: US Public Health Service: Preexposure prophylaxis for the prevention of HIV infection in the United States—2017 Update: a clinical practice guideline. https://www.cdc.gov/hiv/pdf/risk/prep/cdc-hiv-prep-guidelines-2017.pdf. Published March 2018
36

## Slide 37
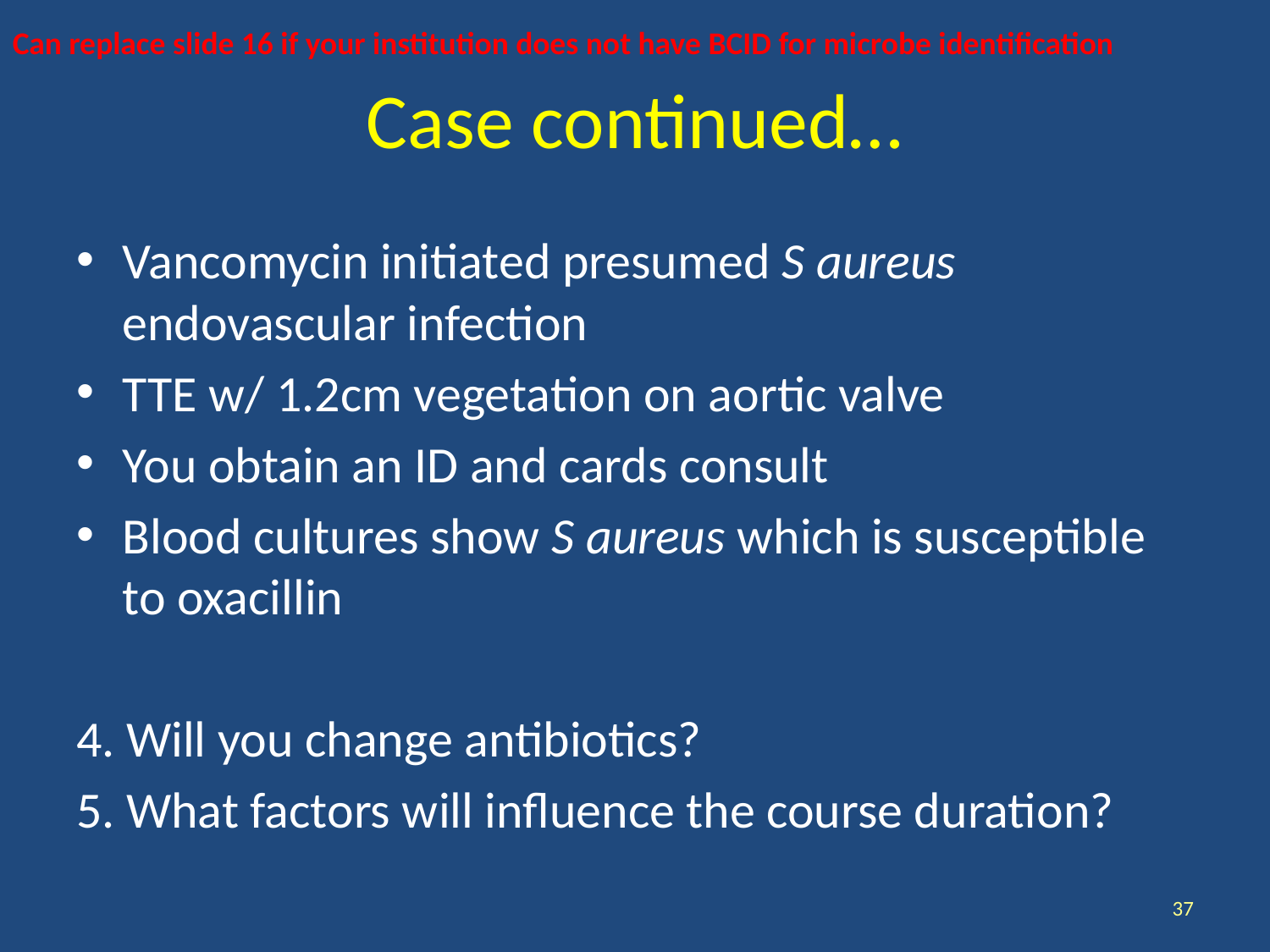

Can replace slide 16 if your institution does not have BCID for microbe identification
# Case continued…
Vancomycin initiated presumed S aureus endovascular infection
TTE w/ 1.2cm vegetation on aortic valve
You obtain an ID and cards consult
Blood cultures show S aureus which is susceptible to oxacillin
4. Will you change antibiotics?
5. What factors will influence the course duration?
37
