# Supplementary material for: Infectious Complications in Injection Drug Use
Source: MedEdPORTAL. 2021 Mar 23;17:11124. doi: 10.15766/mep_2374-8265.11124 (PMC8015638; doi:10.15766/mep_2374-8265.11124)
Supplement: Supplementary file 1 — Facilitator Guide.docxdPre- and Postsurvey.docxInfectious Disease Complications in IDU Workshop.pptxCase 1 and Case 2 Handout.pptxAnswer Key.docx [file mep_2374-8265.11124-s001.zip › D. Case 1 and Case 2 Handout.pptx]

## Slide 1
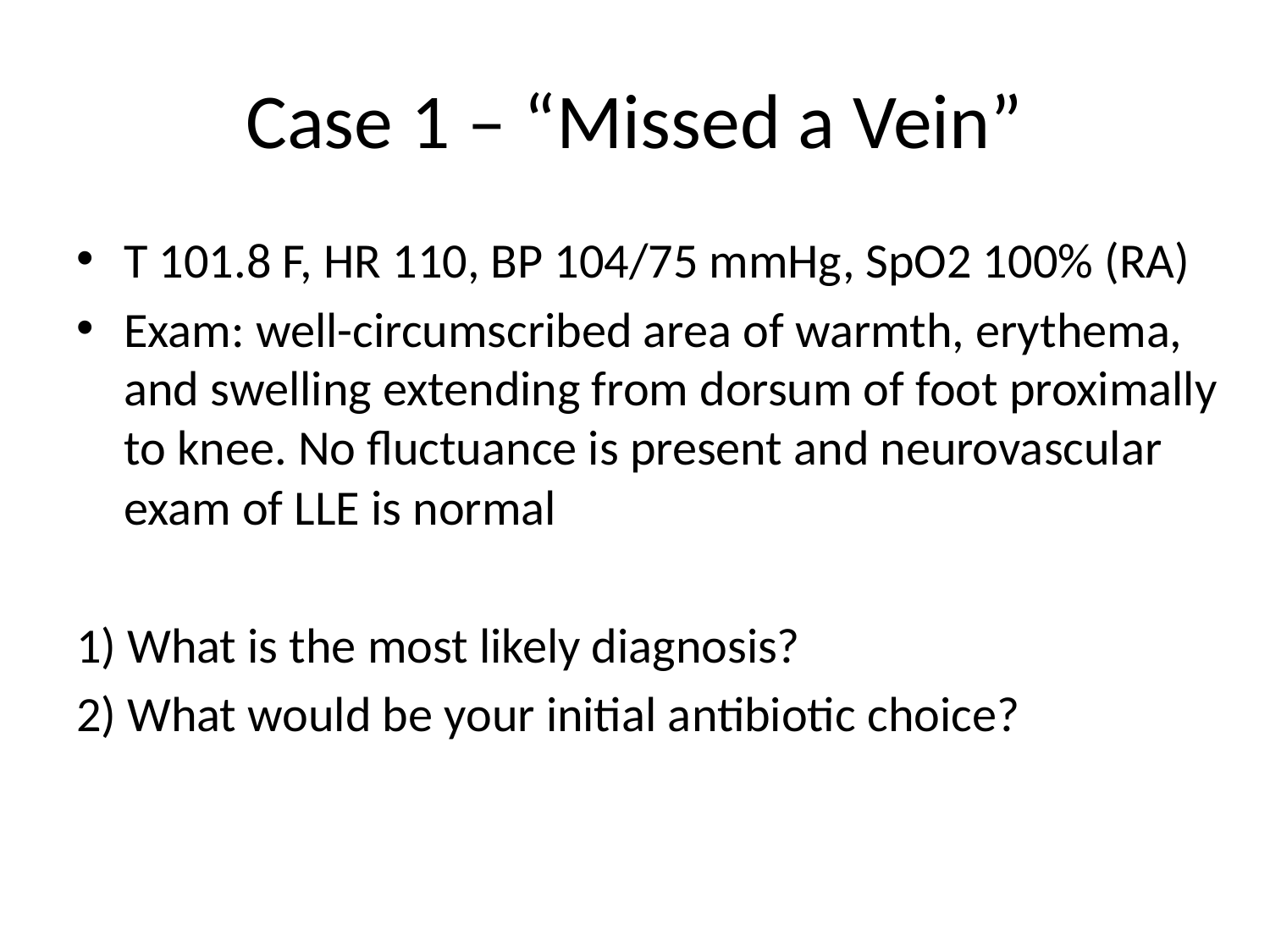

# Case 1 – “Missed a Vein”
T 101.8 F, HR 110, BP 104/75 mmHg, SpO2 100% (RA)
Exam: well-circumscribed area of warmth, erythema, and swelling extending from dorsum of foot proximally to knee. No fluctuance is present and neurovascular exam of LLE is normal
1) What is the most likely diagnosis?
2) What would be your initial antibiotic choice?

## Slide 2
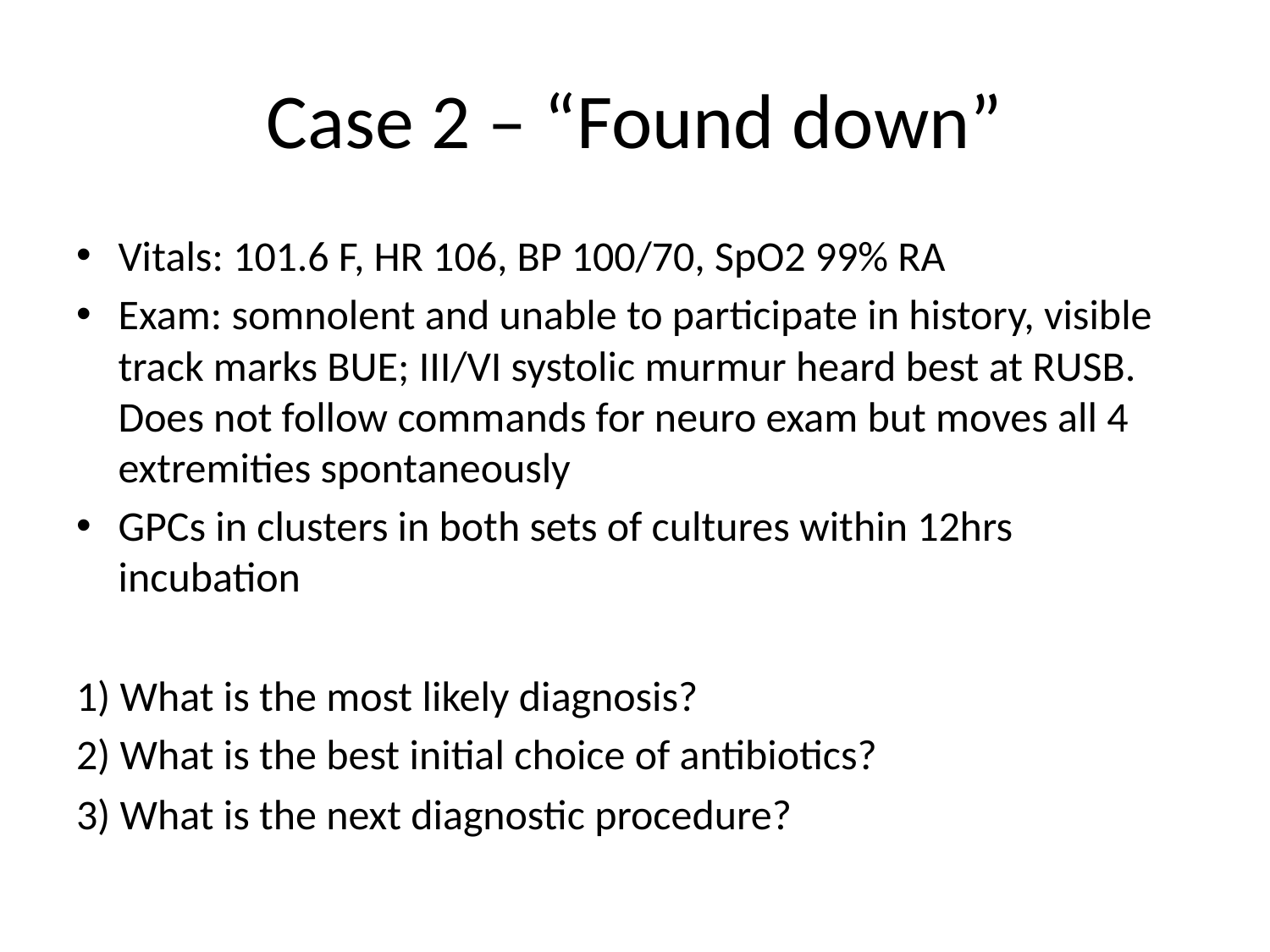

# Case 2 – “Found down”
Vitals: 101.6 F, HR 106, BP 100/70, SpO2 99% RA
Exam: somnolent and unable to participate in history, visible track marks BUE; III/VI systolic murmur heard best at RUSB. Does not follow commands for neuro exam but moves all 4 extremities spontaneously
GPCs in clusters in both sets of cultures within 12hrs incubation
1) What is the most likely diagnosis?
2) What is the best initial choice of antibiotics?
3) What is the next diagnostic procedure?
