# Supplementary material for: Infectious Complications in Injection Drug Use
Source: MedEdPORTAL. 2021 Mar 23;17:11124. doi: 10.15766/mep_2374-8265.11124 (PMC8015638; doi:10.15766/mep_2374-8265.11124)
Supplement: Supplementary file 1 — Facilitator Guide.docxdPre- and Postsurvey.docxInfectious Disease Complications in IDU Workshop.pptxCase 1 and Case 2 Handout.pptxAnswer Key.docx [file mep_2374-8265.11124-s001.zip › E. Answer Key.docx]

**PRE-Survey for “Common Infectious Complications in Injection Drug Use (IDU)”**

*Please complete this short anonymous survey prior to the session.*

Today’s date:______________

PGY year: ________________

Anonymous identification for study purposes: ___________________ (first 2 letters of mother’s maiden name and last 4 digits of your cell phone number)

In the last year, have you participated in the following rotations?

(***Please adapt for clinical experiences available to your learners)

HIV/Infectious Diseases inpatient team Yes No

Infectious Diseases subspecialty clinic Yes No

Infectious Diseases consult service Yes No

1. Of patients you care for in the inpatient setting who inject drugs, how often is the primary reason for admission related to an infectious complication of IDU?

- 1. Never
  2. Less than 25% of the time
  3. 25-50 % of the time
  4. 50-75% of the time
  5. Greater than 75% of the time

2. How comfortable do you feel with your ability to do the following:

(scale of 1-5: where 1 = very uncomfortable, 5= very comfortable). Please circle a response

Clinical Care

| Recognizing the spectrum of severity of SSTIs | 1 | 2 | 3 | 4 | 5 |
| --- | --- | --- | --- | --- | --- |
| Diagnosis and initial management of infective endocarditis | 1 | 2 | 3 | 4 | 5 |
| Diagnosis and early management of osteomyelitis | 1 | 2 | 3 | 4 | 5 |
| Selecting empiric antimicrobial therapies for infectious complications of IDU | 1 | 2 | 3 | 4 | 5 |
| Providing appropriate screening and immunizations for PWID in the outpatient setting | 1 | 2 | 3 | 4 | 5 |
| Identifying PWID in which PrEP is indicated and counseling about its use | 1 | 2 | 3 | 4 | 5 |

Abbreviations: (SSTI = skin and soft tissue infection, PWID = people who inject drugs, PrEP = pre-exposure prophylaxis)

3. A 23-year-old male with history of active injection drug use, treated hepatitis C virus, and depression presents with pain and redness in his forearm after “missing a vein” while injecting the day prior. On exam, his vitals are T 100.9F, HR 92, BP 131/76 mmHg, RR 14, and SpO2 100% on room air. He has well-circumscribed area of warmth, swelling, and erythema just distal to his antecubital fossa with no fluctuance or induration. The decision is made to treat him with oral antibiotics and send him home. Which of the following is the best antimicrobial choice?

a. Cephalexin

b. Amoxicillin-clavulanate

c. Ciprofloxacin

d. Doxycycline

4. A 45-year-old female with history of active injection drug use, HIV well-controlled on antiretroviral therapy (viral load undetectable, CD4 360/31%), and hypertension presents with fevers, chills, and lower back pain. On exam, her vitals are T 102.4F, HR 107, BP 156/101 mmHg, RR 18, and SpO2 99% on room air. She has midline tenderness over the spinous processes of her lower lumbar vertebrae and a normal neuro exam. What is the next most appropriate diagnostic step?

a. Radiographs of lumbar spine

b. MRI lumbar spine

c. CT lumbar spine w/ contrast

d. Transthoracic echocardiogram (TTE)

5. List the screening tests and immunizations (apart from age-appropriate testing/immunizations) that should be applied to PWID in the outpatient setting according to Centers for Disease Control and Prevention.

HIV, HCV, HBV, STI, pregnancy test (screening)

HBV, HAV (immunization)

6. What behaviors of PWID would prompt you as a clinician to initiate a discussion with your patient about pre-exposure prophylaxis (PrEP)?

Sharing needles/equipment

Transactional Sex

**POST-Survey for “Common Infectious Complications in Injection Drug Use”**

*Please complete this short anonymous survey prior to the session.*

Today’s date:______________

PGY year: ________________

Anonymous identification for study purposes: ___________________ (first 2 letters of mother’s maiden name and last 4 digits of your cell phone number)

In the last year, have you participated in the following rotations?

(***Please adapt for clinical experiences available to your learners)

HIV/Infectious Diseases inpatient team Yes No

Infectious Diseases subspecialty clinic Yes No

Infectious Diseases consult service Yes No

1. Of patients you care for in the inpatient setting who inject drugs, how often is the primary reason for admission related to an infectious complication of IDU?

1. Never
2. Less than 25% of the time
3. 25-50 % of the time
4. 50-75% of the time
5. Greater than 75% of the time

2. How comfortable do you feel with your ability to do the following:

(scale of 1-5: where 1 = very uncomfortable, 5= very comfortable). Please circle a response

Clinical Care

| Recognizing the spectrum of severity of SSTIs | 1 | 2 | 3 | 4 | 5 |
| --- | --- | --- | --- | --- | --- |
| Diagnosis and initial management of infective endocarditis | 1 | 2 | 3 | 4 | 5 |
| Diagnosis and early management of osteomyelitis | 1 | 2 | 3 | 4 | 5 |
| Selecting empiric antimicrobial therapies for infectious complications of IDU | 1 | 2 | 3 | 4 | 5 |
| Providing appropriate screening and immunizations for PWID in the outpatient setting | 1 | 2 | 3 | 4 | 5 |
| Identifying PWID in which PrEP is indicated and counseling about its use | 1 | 2 | 3 | 4 | 5 |

Abbreviations: (SSTI = skin and soft tissue infection, PWID = people who inject drugs, PrEP = pre-exposure prophylaxis)

3. A 23-year-old male with history of active injection drug use, treated hepatitis C virus, and depression presents with pain and redness in his forearm after “missing a vein” while injecting the day prior. On exam, his vitals are T 100.9F, HR 92, BP 131/76 mmHg, RR 14, and SpO2 100% on room air. He has well-circumscribed area of warmth, swelling, and erythema just distal to his antecubital fossa with no fluctuance or induration. The decision is made to treat him with oral antibiotics and send him home. Which of the following is the best antimicrobial choice?

a. Cephalexin

b. Amoxicillin-clavulanate

c. Ciprofloxacin

d. Doxycycline

4. A 45-year-old female with history of active injection drug use, HIV well-controlled on antiretroviral therapy (viral load undetectable, CD4 360/31%), and hypertension presents with fevers, chills, and lower back pain. On exam, her vitals are T 102.4F, HR 107, BP 156/101 mmHg, RR 18, and SpO2 99% on room air. She has midline tenderness over the spinous processes of her lower lumbar vertebrae and a normal neuro exam. What is the next most appropriate diagnostic step?

a. Radiographs of lumbar spine

b. MRI lumbar spine

c. CT lumbar spine w/ contrast

d. Transthoracic echocardiogram (TTE)

5. List the screening tests and immunizations (apart from age-appropriate testing/immunizations) that should be applied to PWID in the outpatient setting according to Centers for Disease Control and Prevention.

HIV, HCV, HBV, STI, pregnancy test (screening)

HBV, HAV (immunization)

6. What behaviors of PWID would prompt you as a clinician to initiate a discussion with your patient about pre-exposure prophylaxis (PrEP)?

Sharing needles/equipment

Transactional Sex

7. Was this workshop relevant to your practice? Yes No

8. What aspects of the session were most beneficial?

9. What aspects of the session did you find least valuable and what improvements would you recommend?

10. Give an example of one thing you will do differently because of this session:

11. Would you recommend this session for next year? Yes No
