# Supplementary material for: Infectious Complications in Injection Drug Use
Source: MedEdPORTAL. 2021 Mar 23;17:11124. doi: 10.15766/mep_2374-8265.11124 (PMC8015638; doi:10.15766/mep_2374-8265.11124)
Supplement: Supplementary file 1 — Facilitator Guide.docxdPre- and Postsurvey.docxInfectious Disease Complications in IDU Workshop.pptxCase 1 and Case 2 Handout.pptxAnswer Key.docx [file mep_2374-8265.11124-s001.zip › _Educational_Summary_Report_11124.pdf]

MedEdPORTAL® | The AAMC Journal of Teaching and Learning Resources

Original Publication

Open Access

Infectious Complications in Injection Drug Use

Ryan Knodle, MD*, Lindsay Demers, MS, PhD, Rachel Simmons, MD

*Corresponding author: ryan.knodle@bmc.org

Abstract

Introduction: The prevalence of opioid use disorder has increased steadily over the last decade (from 2.2 million in 2010 to 10.2 million in 2018) and with it, a surge in infectious complications associated with injection drug use (IDU). Trainees in internal medicine routinely diagnose, manage, and treat patients experiencing these infections in the hospital setting as well as screen for and immunize against other comorbid infections in the ambulatory setting. **Methods:** This 90-minute, case-based, interactive workshop was led by two facilitators, an infectious diseases specialist and a senior internal medicine resident. To evaluate its effectiveness, we used a pre- and postsession survey administered at the beginning and end of the workshop. Learners were asked to rate comfort level in recognizing, managing, and counseling about various IDU-related infections, as well as to answer specific, content-level questions. **Result:** Thirty of 42 participants who attended the workshop completed the evaluation. There was a statistically significant change in participants' comfort level with diagnosing and managing IDU-associated infections as well as ambulatory standards of care for people who inject drugs (PWID) from pre- to postworkshop. **Discussion:** Our workshop focused on the management and prevention of infections among PWID in both the inpatient and ambulatory settings. Learners demonstrated increased comfort in managing these conditions.

Keywords

Injection Drug Use, Skin and Soft Tissue Infections, Infective Endocarditis, Pre-Exposure Prophylaxis, Endovascular Infection, Opioid Use Disorder, Substance Abuse/Addiction, Case-Based Learning, Opioids, Addiction, Pain

Educational Objectives

By the end of the workshop, learners will increase comfort in their ability to:

1. Identify the spectrum of skin and soft tissue infections related to injection drug use and select an appropriate antimicrobial regimen.
2. Describe clinical findings consistent with infective endocarditis (IE) and demonstrate initial management plans regarding antimicrobial therapy and further diagnostics.
3. Generate a differential diagnosis for acute back pain in patients with IE/endovascular infections.
4. List screening tests and immunizations unique to people who inject drugs (PWID).
5. Identify PWID who are appropriate candidates for pre-exposure prophylaxis and provide appropriate counseling regarding risks, benefits, and efficacy.

Citation:
Knodle R, Demers L, Simmons R. Infectious complications in injection drug use. *MedEdPORTAL*. 2021;17:11124.
https://doi.org/10.15766/mep_2374-8265.11124

Introduction

The rate of opioid use disorder (OUD) in the US has been steadily increasing over the last 2 decades, from 1.7 million in 2002 to 2.2 million in 2010 to a staggering 10.2 million in 2018.[1-4] Along with increased incidence of injection drug use (IDU) among communities, there has been a concomitant rise in infections related to IDU, resulting in higher utilization of health care: both visits to the emergency department and inpatient admissions for more serious infections. Over a 10-year period, there was a 72% rise in hospitalizations related to OUD and a near doubling of admissions for serious infections (from 3,421 in 2002 to 6,535 in 2012). This included a 1.5-fold increase in infective endocarditis (IE), a 2.2-fold increase in osteomyelitis, and a 2.6-fold increase in spinal epidural abscesses.[5] From 1993 to 2010, the national incidence of skin and soft tissue infections related to opioid use doubled (from fewer than four per 100,000 to over nine per 100,000).[6,7] From a financial perspective, Medicaid costs associated with the opioid epidemic at large are estimated to have quadrupled from two billion to eight billion dollars between 1999 and 2013.[8]

Infections related to IDU are becoming uncomfortably routine for internal medicine trainees and practitioners to diagnose and

Copyright © 2021 Knodle et al. This is an open-access publication distributed under the terms of the Creative Commons Attribution license.

manage in the hospital; additionally, the spectrum and severity of these disease processes can be quite broad. Studies show that more than 60% of internal medicine residents feel unprepared to manage substance use disorders (SUDs) broadly despite the fact that people struggling with these disorders make up over one-quarter of the patients they treat.[9] Many internists are also responsible for continued care of these patients in the ambulatory setting, after they leave the hospital. Thus, discussions surrounding risk reduction, screening tests, and tailored immunizations are crucial to promoting health and reducing morbidity/mortality in people who inject drugs (PWID).

Although there has been an expansion in *MedEdPORTAL* content regarding safe prescribing of opioid pain medications, as well as in modules training students and residents how to have difficult conversations regarding pain control and dependence, there are limited educational resources addressing the infectious diseases associated with IDU.[10-13] Similarly, although there are recent *MedEdPORTAL* publications about pre-exposure prophylaxis (PrEP) education, these largely concentrate on indications based on high-risk sexual activity and not IDU.[14,15] Our workshop is unique in its focus on screening, prevention, diagnosis, and treatment of infectious complications of IDU in diverse care settings. We have drawn on pertinent guidelines from various medical specialties to support the workshop. Some aspects referenced in the workshop but not covered in detail include initiation of medications for OUD, which was discussed at length in a joint module on HIV and addiction,[16] as well as education on taking a thorough and complete SUD history and harm reduction/infection prevention.

Methods

We designed this workshop for the residents in our internal medicine program, located within a large academic medical center in a major metropolitan area. We specifically targeted the interns at the end of their first year of training in order to explore these topics after they had clinical exposure on the wards, in the emergency department, and in the outpatient setting. The workshop could also be appropriate for advanced medical students (in their clinical rotations), junior and senior medicine residents, and interns/residents in family medicine or preventive medicine. Prior to implementation, the workshop was reviewed by the Boston Medical Center Institutional Review Board and deemed to be exempt.

Our learners were familiar with the basic experiences of PWID given their training at a safety-net hospital and caring for a large volume of patients struggling with SUDs. For learners who may not have had the same experiences, we recommend reviewing the article by Trowbridge and colleagues for appropriate background knowledge.[17]

We hosted this session during protected lecture time within the ambulatory care block. Unless prior arrangements were made, attendance at the session was expected, but completion of the survey was optional. This session was approximately 90 minutes in total, but it could be adapted for a shorter period by leaving out parts of case 2 (either the section on back pain, the section on the partial oral treatment of endocarditis [POET] study, or both) or the entirety of case 3. The workshop was designed to be facilitated by an infectious disease specialist or a general internist. For our sessions, the facilitators were a PGY 3 internal medicine resident and an infectious disease attending, both of whom treated patients in the inpatient and outpatient settings. The workshop was conducted in person for two sessions and virtually for two sessions given constraints associated with COVID-19.

The cases in the workshop were designed to utilize small-group discussion followed by large-group discussion with the facilitator. They could also be adapted solely to use large-group discussion.

Workshop Outline

The workshop was divided into five sections, which are described in more detail below as well as in the facilitator guide (Appendix A), plus presession and postsession surveys:

- Administration of presession survey: 5 minutes.
- Introduction and objectives: 5 minutes.
- Case 1 (both small- and large-group elements): 20 minutes.
- Case 2 (both small- and large-group elements): 35 minutes.
- Case 3 (optional small- and large-group elements): 15 minutes.
- Summary of learning points and wrap-up: 5 minutes.
- Administration of postsession survey: 5 minutes.

Introductions and objectives: Both facilitators introduced themselves at the beginning of the workshop and led brief introductions for the participants. We had the audience complete an anonymous presession survey (Appendix B) prior to the workshop. We presented the learning objectives using the PowerPoint presentation (Appendix C) and divided the participants into groups of three or four for subsequent small-group activities. Given that we had between nine and 13 participants for each session, we always had at least three small groups. Clinical summary slides (Appendix D) were printed

Copyright © 2021 Knodle et al. This is an open-access publication distributed under the terms of the Creative Commons Attribution license.

prior to the session to be distributed to each participant for the discussions of cases 1 and 2.

Case 1: Case 1 featured a patient with nonpurulent cellulitis. We asked participants to answer questions about the diagnosis and the empiric antimicrobial regimen. We then emphasized the Infectious Diseases Society of America guideline about coverage of methicillin-resistant Staphylococcus aureus in PWID.[18]

Case 2: Case 2 featured a patient with mitral valve IE. We spent time discussing interview questions and physical exam findings for IE, odds ratios presented in the literature, and an introduction to the modified Duke criteria. We asked participants to identify methicillin-susceptible Staphylococcus aureus based on microbiologic data and to select appropriate narrowing and duration of antimicrobial therapy. We also had the audience generate a differential diagnosis for acute-onset back pain in PWID. We concluded with an examination of the POET study, asking participants to determine the patient in case 2's eligibility.[19]

Case 3: Case 3 featured a patient with active IDU presenting to establish care, and we reviewed appropriate screening and prevention for PWID. Participants were asked to generate a list of screening tests as well as immunizations the patient was eligible for. We considered the indications for PrEP in PWID and reviewed practical counseling tips for initiating PrEP.

Summary of learning points/wrap-up: We closed by delineating the learning objectives and key take-home points for the three cases and opened discussion for any further questions.

Evaluation tool: We chose paired presession and postsession surveys (Appendix B, with answer key in Appendix E) to evaluate the impact and effectiveness of our workshop. Participants created a unique identifier for pairing purposes while simultaneously preserving anonymity of responses. The survey contained questions to identify previous experiences with infectious complications of IDU (inpatient infectious diseases ward team, inpatient infectious diseases consult, or outpatient infectious diseases clinic) and also attempted to quantify a participant's estimate of the frequency with which IDU-related complications were treated in our hospital. To subjectively measure an individual's comfort level with several topics covered in the workshop, we used a 5-point Likert scale (1 = *very uncomfortable,* 5 = *very comfortable*) to obtain a baseline measurement as well as a postworkshop measurement. We also included two multiple-choice and two short-answer questions based on content included in the workshop to have an objective measure of effectiveness of material. These questions were developed from our learning objectives and from the three cases presented in the workshop; they were not piloted prior to implementation.

Room Setup, Equipment, and Environment

For the in-person sessions, we used a large conference room with a boardroom-style table in the center. The room was equipped with a large smartboard, which displayed the PowerPoint presentation. There was also a whiteboard in the room for listing responses as detailed above if needed. For the virtual sessions, we had all participants join the same videoconference call hosted by the presenter remotely via their own electronic devices. The presenter had the ability to share the contents of their screen with the audience, driving the PowerPoint presentation from their own laptop.

Results

Forty-two internal medicine PGY 1 residents participated in our workshop, 30 of whom took part in the evaluation. The 12 who were not included completed either the pre- or postsession survey but not both. In order to assess pre- to postworkshop changes in participants' responses to the multiple-choice and short-answer questions on content covered in the workshop, as well questions regarding their comfort level with recognizing, diagnosing, and managing a variety of infectious complications associated with IDU, we used nonparametric sign tests appropriate for Likert-type data. As shown in the Table, there was a statistically significant change in all questions. There was a positive increase in each of the six questions asking about levels of comfort in diagnosing and managing infectious complications of IDU as well as in the two multiple-choice questions.

For the two short-answer questions, we observed an increase in the number of correct answers provided (average: 5.5 vs. 3.6 out of seven possible correct answers, $p \leq .001$). The majority of initial answers for the question regarding screening tests and immunizations for PWID focused on viral hepatitis testing (HIV, hepatitis B, hepatitis C) but became much more diverse following the session. Similarly, while sharing equipment was universally recognized as a criterion for PrEP eligibility before the session, the group's understanding of transactional sex as the other risk factor increased after the session, and more participants answered correctly on the post- compared to the presession survey (93% vs. 30%, $p \leq .001$).

Discussion

Our workshop fills a meaningful gap in *MedEdPORTAL* focusing on the opioid epidemic by directly addressing the infectious

Copyright © 2021 Knodle et al. This is an open-access publication distributed under the terms of the Creative Commons Attribution license.

Table. Change in Pre- and Postsurvey Participant Responses ($N = 30$)

Question[a]	*M (SD)* Pre	*M (SD)* Post	Negative Differences	Ties	Positive Differences
How comfortable do you feel with your ability to do the following:					
Recognizing the spectrum of severity of SSTIs.	3.4 (0.6)	4.2 (0.8)	1	7	22[b]
Diagnosis and initial management of infective endocarditis.	3.6 (0.8)	4.5 (0.6)	1	7	22[b]
Diagnosis and early management of osteomyelitis.	3.4 (0.8)	4.1 (0.7)	2	11	17[b]
Selecting initial antimicrobial therapies for infectious complications of IDU.	3.0 (0.7)	4.3 (0.6)	0	5	25[b]
Providing appropriate screening and immunizations for PWID in the outpatient setting.	2.6 (1.1)	4.4 (0.6)	0	3	27[b]
Identifying PWID in which PrEP is indicated and counseling about its use.	2.5 (1.0)	4.2 (0.7)	1	3	26[b]

Abbreviations: IDU, injection drug use; PrEP, pre-exposure prophylaxis; PWID, people who inject drugs; SSTI, skin and soft tissue infection.
[a]Rated on a 5-point scale (1 = *very uncomfortable,* 5 = *very comfortable*).
[b]Significant at $p \leq .001$.

complications of IDU. It is the only workshop to date that highlights infectious complications commonly encountered by medical students and house staff. As the epidemic continues, it is clear that learners will continue to manage PWID in many different settings; our workshop balances this by providing instruction on aspects related to both inpatient care and the ambulatory setting. Despite focusing on infectious complications of IDU, we also feel strongly about the importance of treating underlying addiction and comorbid psychiatric disease, as well as counseling patients on harm reduction behavior, including safe injection practices. Although we did not have time to cover them in this workshop, this should not undervalue the importance of these topics.

The workshop achieved its intended purpose of improving the participants' comfort level in diagnosing and managing a variety of infectious complications of IDU as well as developing a foundation of knowledge on some basics of care. The content is broadly generalizable and can have an impact on learners from multiple specialties, including internal medicine and family medicine, as well being applicable to senior medical students with some modification. We attribute the workshop's success to its practical, case-based approach to teaching key diagnostic and management tenets, as well as its incorporation of both small- and large-group engagement.

A lesson we learned was how to conduct the workshop virtually as changes in social distancing guidelines due to COVID-19 allowed us to conduct the first two sessions in person but dictated that the last two be hosted remotely. We did this via teleconferencing software using the screen share feature. We did not utilize the small-group feature (breakout rooms) of this software, but if the workshop is conducted virtually in the future, the breakout room feature would be a useful tool to allow small groups to meet and discuss before convening the large group. We also learned that consulting an expert in our local opioid epidemic was critical to sharing pragmatic directives about local disease trends with our audience. This expert provided important information that enhanced the interview questions discussed in case 3, incorporating local use trends into nonjudgmental conversations.

There are a few limitations to this project. First, although the content was applicable to our residents given the patient population they cared for, it may not be generalizable to other programs with markedly different patient populations or low incidence of PWID. Second, the session was conducted at a single institution. Third, our postworkshop evaluation tool was administered immediately following completion of the workshop and thus was unable to assess for long-term retention or efficacy. Fourth, our outcomes were all knowledge based, not practice based, making it difficult to assess through the lens of patient outcomes.

In the future, we would like to continue to develop curricula focusing on IDU, specifically on both inpatient and outpatient treatment with medications for OUD, as well as safe injection practices for harm reduction. We would also like to expand content regarding taking a thorough and sensitive substance use history.

Appendices

A. Facilitator Guide.docx

B. Pre- and Postsurvey.docx

C. Infectious Disease Complications in IDU Workshop.pptx

D. Case 1 and Case 2 Handout.pptx

E. Answer Key.docx

All appendices are peer reviewed as integral parts of the Original Publication.

Copyright © 2021 Knodle et al. This is an open-access publication distributed under the terms of the Creative Commons Attribution license.

Ryan Knodle, MD: Resident, Department of Medicine, Boston University School of Medicine; ORCID: https://orcid.org/0000-0002-8703-9658

Lindsay Demers, MS, PhD: Assistant Professor, Department of Medicine, Boston University School of Medicine

Rachel Simmons, MD: Associate Residency Program Director and Assistant Professor, Section of Infectious Diseases, Department of Medicine, Boston University School of Medicine

Disclosures
None to report.

Funding/Support
None to report.

Ethical Approval
The Boston Medical Center Institutional Review Board approved this study.

References

1. Centers for Disease Control and Prevention. *2019 Annual Surveillance Report of Drug-Related Risks and Outcomes—United States Surveillance Special Report*. Centers for Disease Control and Prevention, U.S. Department of Health and Human Services; 2019. Accessed June 1, 2020. https://www.cdc.gov/drugoverdose/pdf/pubs/2019-cdc-drug-surveillance-report.pdf

2. Substance Abuse and Mental Health Services Administration. *Results From the 2010 National Survey on Drug Use and Health: Summary of National Findings*. Substance Abuse and Mental Health Services Administration; 2011. HHS publication (SMA) 11-4658. Accessed June 1, 2020. https://www.samhsa.gov/data/sites/default/files/NSDUHNationalFindingsResults2010-web/2k10ResultsRev/NSDUHresultsRev2010.pdf

3. Substance Abuse and Mental Health Services Administration. *Results From the 2002 National Survey on Drug Use and Health: National Findings*. Substance Abuse and Mental Health Services Administration; 2003. DHHS publication SMA 03-3836. Accessed June 1, 2020. https://files.eric.ed.gov/fulltext/ED479833.pdf

4. Rudd RA, Aleshire N, Zibbell JE, Gladden RM. Increases in drug and opioid overdose deaths—United States, 2000-2014. *MMWR Morb Mortal Wkly Rep*. 2016;64(50-51):1378-1382. https://doi.org/10.15585/mmwr.mm6450a3

5. Ronan MV, Herzig SJ. Hospitalizations related to opioid abuse/dependence and associated serious infections increased sharply, 2002–12. *Health Aff (Millwood)*. 2016;35(5):832-837. https://doi.org/10.1377/hlthaff.2015.1424

6. Ciccarone D, Unick GJ, Cohen JK, Mars SG, Rosenblum D. Nationwide increase in hospitalizations for heroin-related soft tissue infections: associations with structural market conditions. *Drug Alcohol Depend*. 2016;163:126-133. https://doi.org/10.1016/j.drugalcdep.2016.04.009

7. Mori M, Brown KJ, Bin Mahmood SU, Geirsson A, Mangi AA. Trends in infective endocarditis hospitalizations, characteristics, and valve operations in patients with opioid use disorders in the United States: 2005-2014. *J Am Heart Assoc*. 2020;9(6):e012465. https://doi.org/10.1161/JAHA.119.012465

8. Leslie DL, Ba DM, Agbese E, Xing X, Liu G. The economic burden of the opioid epidemic on states: the case of Medicaid. *Am J Manag Care*. 2019;25(13 suppl):S243-S249. https://pubmed.ncbi.nlm.nih.gov/31361426/

9. Wakeman SE, Baggett MV, Pham-Kanter G, Campbell EG. Internal medicine residents' training in substance use disorders: a survey of the quality of instruction and residents' self-perceived preparedness to diagnose and treat addiction. *Subst Abus*. 2013;34(4):363-370. https://doi.org/10.1080/08897077.2013.797540

10. Baxter J. Minimizing the misuse of prescription opioids in patients with chronic non-malignant pain. *MedEdPORTAL*. 2012;8:9205. https://doi.org/10.15766/mep_2374-8265.9205

11. Klapheke M, Pasarica M. Opioid risk mitigation strategies and overdose resuscitation. *MedEdPORTAL*. 2017;13:10621. https://doi.org/10.15766/mep_2374-8265.10621

12. Vettese TE, Thati N, Roxas R. Effective chronic pain management and responsible opioid prescribing: aligning a resident workshop to a protocol for improved outcomes. *MedEdPORTAL*. 2018;14:10756. https://doi.org/10.15766/mep_2374-8265.10756

13. Sagin A, Kimberly SM, Farabelli JP, Schafer K, Kumar P, Uritsky TJ. Teaching pain management in serious illness in the era of the opioid epidemic: a team-based intervention. *MedEdPORTAL*. 2020;16:11006. https://doi.org/10.15766/mep_2374-8265.11006

14. Bunting SR, Saqueton R, Batteson TJ. A guide for designing student-led, interprofessional community education initiatives about HIV risk and pre-exposure prophylaxis. *MedEdPORTAL*. 2019;15:10818. https://doi.org/10.15766/mep_2374-8265.10818

15. Perucho J, Alzate-Duque L, Bhuiyan A, Sánchez JP, Sánchez NF. PrEP (pre-exposure prophylaxis) education for clinicians: caring for an MSM patient. *MedEdPORTAL*. 2020;16:10908. https://doi.org/10.15766/mep_2374-8265.10908

16. Simmons R, Siegel J. Introduction to HIV and addiction medicine for residents: integrated case-based learning. *MedEdPORTAL*. 2016;12:10375. https://doi.org/10.15766/mep_2374-8265.10375

17. Trowbridge P, Weinstein ZM, Kerensky T, et al. Addiction consultation services—linking hospitalized patients to outpatient

Copyright © 2021 Knodle et al. This is an open-access publication distributed under the terms of the Creative Commons Attribution license.

addiction treatment. *J Subst Abuse Treat.* 2017;79:1-5. https://doi.org/10.1016/j.jsat.2017.05.007

18. Stevens DL, Bisno AL, Chambers HF, et al. Practice guidelines for the diagnosis and management of skin and soft tissue infections: 2014 update by the Infectious Diseases Society of America. *Clin Infect Dis*. 2014;59(2):e10-e52. https://doi.org/10.1093/cid/ciu296

19. Iversen K, Ihlemann N, Gill SU, et al. Partial oral versus intravenous antibiotic treatment of endocarditis. *N Engl J Med*. 2019;380(5):415-424. https://doi.org/10.1056/NEJMoa1808312

Received: August 3, 2020
Accepted: January 12, 2021
Published: March 23, 2021

Copyright © 2021 Knodle et al. This is an open-access publication distributed under the terms of the Creative Commons Attribution license.
